# Spatiotemporal dynamics of fetal liver hematopoietic niches

**DOI:** 10.1084/jem.20240592

**Published:** 2025-01-07

**Authors:** Márcia Mesquita Peixoto, Francisca Soares-da-Silva, Valentin Bonnet, Yanping Zhou, Gustave Ronteix, Rita Faria Santos, Marie-Pierre Mailhe, Gonçalo Nogueira, Xing Feng, João Pedro Pereira, Emanuele Azzoni, Giorgio Anselmi, Marella F.T.R. de Bruijn, Archibald Perkins, Charles N. Baroud, Perpétua Pinto-do-Ó, Ana Cumano

**Affiliations:** 1 Immunology Department, Unit of Lymphocytes and Immunity, Institut Pasteur, Paris, France; 2 INSERM U1223, Paris, France; 3 i3S – Instituto de Investigação e Inovação em Saúde & INEB – Instituto Nacional de Engenharia Biomédica, Universidade do Porto, Porto, Portugal; 4 Abel Salazar Biomedical Sciences Institute, University of Porto, Porto, Portugal; 5 Université Paris Cité, Cellule Pasteur, Paris, France; 6 Physical Microfluidics and Bioengineering, Institut Pasteur, Université Paris Cité, Paris, France; 7 Laboratoire d’Hydrodynamique, Centre National de la Recherche Scientifique, École Polytechnique, Institut Polytechnique de Paris, Palaiseau, France; 8 Department of Immunobiology and Yale Stem Cell Center, Yale University School of Medicine, New Haven, CT, USA; 9 School of Medicine and Surgery, University of Milano-Bicocca, Monza, Italy; 10 Radcliffe Department of Medicine, MRC Molecular Haematology Unit, MRC Weatherall Institute of Molecular Medicine, University of Oxford, Oxford, UK; 11 Department of Pathology and Laboratory Medicine, University of Rochester Medical Center, Rochester, NY, USA

## Abstract

Embryonic hematopoietic cells develop in the fetal liver (FL), surrounded by diverse non-hematopoietic stromal cells. However, the spatial organization and cytokine production patterns of the stroma during FL development remain poorly understood. Here, we characterized and mapped the hematopoietic and stromal cell populations at early (E12.5–14.5) FL stages, revealing that while hepatoblasts were the primary source of hematopoietic growth factors, other stromal cells—including mesenchymal, mesothelial, and endothelial cells—also contributed to this signaling network. Using a dedicated image analysis pipeline, we quantified cell distances to tissue structures and defined neighbor relationships, uncovering that different hematopoietic progenitors exhibit distinct preferences for neighboring stromal cells and show developmental changes in spatial distribution. Notably, our data suggest that the sub-mesothelium region plays a prominent role in early fetal hematopoiesis. This approach offers a valuable tool for studying complex cellular interactions in biological systems, providing new insights into hematopoietic niche organization during development.

## Teaser

This work shows that hepatoblasts in the fetal liver are the primary source of factors that sustain blood cell production. Different hematopoietic progenitors exhibit distinct preferences for neighboring stromal cells and show developmental changes in spatial distribution.

## Introduction

The generation of hematopoietic cells occurs in different embryonic locations at successive, overlapping, time windows (Elsaid et al., 2020; Soares-da-Silva et al., 2023). In the mouse, primitive erythrocytes are first produced within the yolk sac (YS) blood islands, after embryonic day (E) 7 of gestation (Palis, 2016). Later, between E8 and E8.5, multipotent erythro-myeloid progenitors (EMPs) are produced through an endothelial-to-hematopoietic transition (EHT) process in the vasculature of the YS (Frame et al., 2016; Kasaai et al., 2017; McGrath et al., 2015). Starting at E9 and spanning for around 48 h, a subset of endothelial cells in the intraembryonic (IE) vasculature (dorsal aorta, and umbilical and vitelline arteries) undergoes EHT, resulting in the emergence of the first lympho-erythro-myeloid progenitors (Cumano et al., 1996; de Bruijn et al., 2000; Godin et al., 1999; Medvinsky and Dzierzak, 1996; Yokomizo et al., 2011; Yokomizo and Dzierzak, 2010). These newly generated multipotent progenitors (MPPs) can undergo expansion *in situ* but rapidly enter the circulation and home to the fetal liver (FL) (Rybtsov et al., 2016; Taoudi et al., 2008), where cells from all three hematopoietic waves converge. The FL then becomes a hematopoietic hub, where a subset of MPPs acquires long-term reconstitution capacity and more committed progenitors promptly fulfill the embryo requirements (Kieusseian et al., 2012; Medvinsky and Dzierzak, 1996; Soares-da-Silva et al., 2021).

The FL is a highly dynamic organ undergoing continuous and profound changes throughout development. Hepatoblasts, of endodermal origin, are bipotent epithelial progenitors that later differentiate into hepatocytes and cholangiocytes, the main functional units of the adult liver (Zorn, 2008). Mesothelial cells (MCs) derived from the septum transversum form the mesothelium, an epithelial layer covering the surface of the liver. A subset of these cells undergoes epithelial-to-mesenchymal transition (EMT) and migrate inward from the FL surface to produce hepatic stellate cells (SCs, also called pericytes), portal fibroblasts (PFs), and smooth muscle cells (Lua and Asahina, 2016). Three major mesenchymal liver populations can be considered according to their location: sub-MCs (or capsular fibroblasts), adjacent to MCs (Lua and Asahina, 2016); SCs, around sinusoids, that mature into vitamin A storing cells (Kubota et al., 2007; Peixoto et al., 2022); and NG2^+^ PFs found exclusively around portal vessels (Khan et al., 2016). These populations have not been well defined for their molecular signature, and little is known about their functional differences. After E16.5, the liver transits from a hematopoietic to a metabolic organ and hepatoblasts rapidly differentiate into hepatocytes and cholangiocytes, the latter dependent on signals from NG2^+^ PFs around the portal vessels, for differentiation (Zorn, 2008).

The pioneer stem cell niche hypothesis early advanced by Schofield suggested that stem cells’ behavior is determined by their distinct spatial placement, effectively preventing their maturation and ensuring continuous maintenance (Schofield, 1978). Multiple bone marrow (BM) stromal populations namely endothelial, osteoblasts, and mesenchymal cells, have been proposed as hematopoietic regulators (Crane et al., 2017; Morrison and Scadden, 2014). A recent study performed an extensive quantitative analysis regarding the location of hematopoietic stem cells (HSCs) in relation to multiple stromal populations in the adult BM (Kokkaliaris et al., 2020). They found HSCs to be in close/direct contact with sinusoids, CAR cells, and megakaryocytes (Mks), but not with bone, adipocyte, periarteriolar, or Schwann cells. However, the distance of HSCs to all cell types assessed did not differ from a randomized scenario, suggesting that HSCs do not occupy a specific position within the BM.

Although the FL microenvironment has received much less attention than the BM, multiple FL cell types have been reported to play regulatory roles in fetal hematopoiesis at the progenitors level (maintenance or expansion) and/or supporting their differentiation toward distinct lineages (Soares-da-Silva et al., 2020). DLK1-expressing cells (considered as hepatoblasts), endothelial cells, SCs, and NG2^+^ PFs cells were found to express hematopoietic cytokines and were assumed to be essential for HSC maintenance (Chou et al., 2013; Chou and Lodish, 2010; Khan et al., 2016; Lee et al., 2021). However, removal of NG2^+^ PFs and constitutive deletion of Kit ligand (KITL) in hepatoblasts, endothelial, or SCs resulted in none or subtle reductions in the HSC compartment before birth (Khan et al., 2016; Lee et al., 2021). Of note, most studies restricted their analysis to particular subsets and/or concentrated in single time points, not considering the early stages of FL hematopoiesis, and not comparing cytokine production by different stromal subsets (Iwasaki et al., 2010; Khan et al., 2016; Lee et al., 2021). Therefore, a comprehensive analysis of the relative contribution of the different stromal components to hematopoietic cytokine production and their localization relative to the different hematopoietic populations along development is still lacking.

Here, we performed an extensive characterization of the FL hematopoietic niche by flow cytometry (FC), transcriptional signature profiling, and immunofluorescence (IF) during development. We developed an imaging strategy of FL sections for the analysis of different stromal/hematopoietic cell populations and implemented a deep learning-based image analysis pipeline to segment and classify individual cells and to describe their spatial distribution and immediate neighbors. We identified the cytokine producer cells in the FL throughout development, reporting an unidentified role of MCs as producers of KITL, and used the *Cxcl12*-dsRed and *Kitl*-tdTomato models to visualize the location of two major hematopoietic regulators in the FL. We observed non-random distributions of different hematopoietic cell types, with higher frequencies of YS- and IE-derived progenitors at the vicinity of the sub-mesothelium region at E12.5 (∼50 μm away from the liver’s surface, here referred as periphery), which are maintained at E14.5 for YS (CXCR4^−^) cells, but no longer for IE (CXCR4^+^) cells, likely due to their differences in response to CXCL12. Throughout development, hematopoietic progenitors preferentially neighbored hepatoblasts, which appeared as the cells producing the most diverse array of cytokines. Using a spheroid 3D assay, we showed that hepatoblasts alone ensure the expansion of lineage-negative, KIT^+^Sca-1^+^ (LSK) cells and that KITL is the factor that mediates that expansion. At E14.5, we identified a subset of stem and early progenitor cells that comprise LSK cells, 40% of which are CD48^−^. These cells were identified as YFP^+^ in Mds1^CreERT2 Rosa26YFP^ mice and represent the founders of the adult HSC compartment (Zhang et al., 2021). These early progenitors do not neighbor any stroma cell type at frequencies higher than expected by their relative frequencies, suggesting that adult HSCs are randomly distributed throughout the FL parenchyma.

## Results

### The non-hematopoietic compartment of the FL comprises endothelial, mesothelial, mesenchymal, and hepatic cells

**Figure 1.**
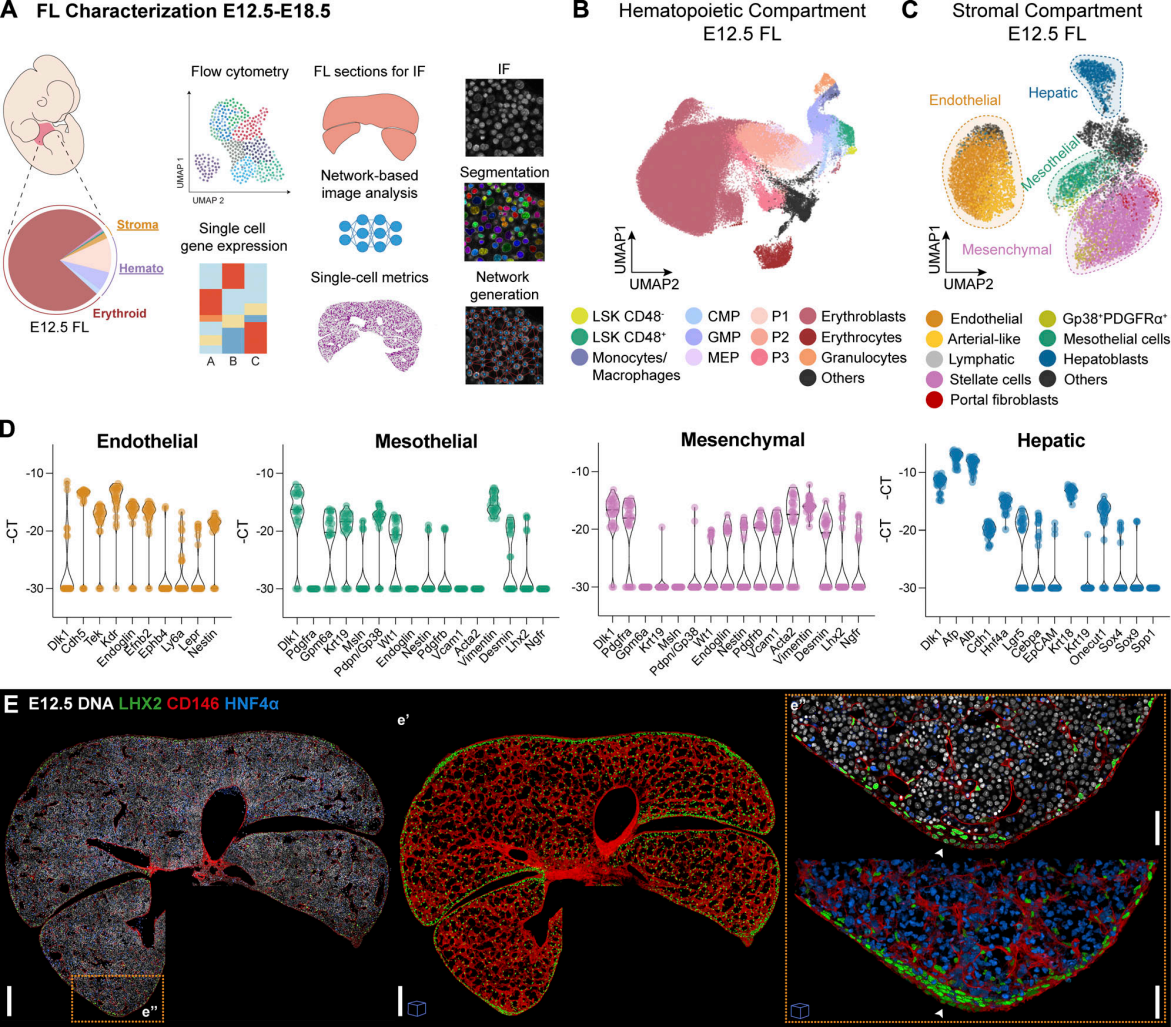
**The non-hematopoietic compartment of the FL comprises endothelial, mesothelial, mesenchymal, and hepatic cells. (A)** Schematic representation of the devised strategy for the analysis of the FL from E12.5 to E18.5. **(B)** UMAP analysis of FC data of E12.5 FLs stained with the surface markers Ter119, CD45, CD71, Gr1, CD11b, KIT, Sca-1, CD16/32, CD34, CD150, and CD48. See gating strategy used in Fig. S1 A. **(C)** UMAP analysis of FC data of Ter119^−^CD45^−^CD71^−^KIT^−^ cells from E12.5 FLs stained with the surface markers CD146, CD31, Gp38, CD54, CD51, Thy1.2, PDGFRα, NG2, ALCAM, Sca-1, E-Cadh, and EpCAM that identify the four major populations highlighted by dashed circles: endothelial, hepatic, mesothelial, and mesenchymal. See the gating strategy used in Fig. S1 B. **(D)** Violin plots of single-cell gene expression analysis of lineage associated transcripts of sorted endothelial (CD31^+^CD146^+^, orange), mesothelial (CD31^−^PDGFRα^−^Gp38^+^, green), mesenchymal (CD31^−^Gp38^−^PDGFRα^+^, pink), and hepatic (CD31^−^Gp38^−^PDGFRα^−^EpCAM^−^E-Cadh^+^, blue) cells from E12.5 FLs. Each dot represents a cell. **(E)** Representative single-stack IF of an E12.5 FL section with DNA (white), LHX2 (green), CD146 (red), and HNF4α (blue). Scale bar, 200 μm. **(e′)** Insert e′ shows a 3D view (40 μm projection) of the same FL section showing only CD146 and LHX2. **(e″) **Insert e″ represents enlarged single-stacks and a 3D view (40 μm projection) of the selected regions. White arrowheads point to MCs. Images with cubes are 3D projections. Scale bars, 50 μm.

While the FL plays a crucial role in embryonic hematopoiesis, the specific functions of stromal subsets within this organ remain poorly understood. Here, we identified and characterized the hematopoietic and stromal compartments using FC and single-cell transcriptional profiling of isolated FL cells. We coupled this single-cell approach with an imaging pipeline to analyze and quantify the spatial organization of the main hematopoietic and stromal populations at the cellular level (Fig. 1 A).

**Figure S1.**
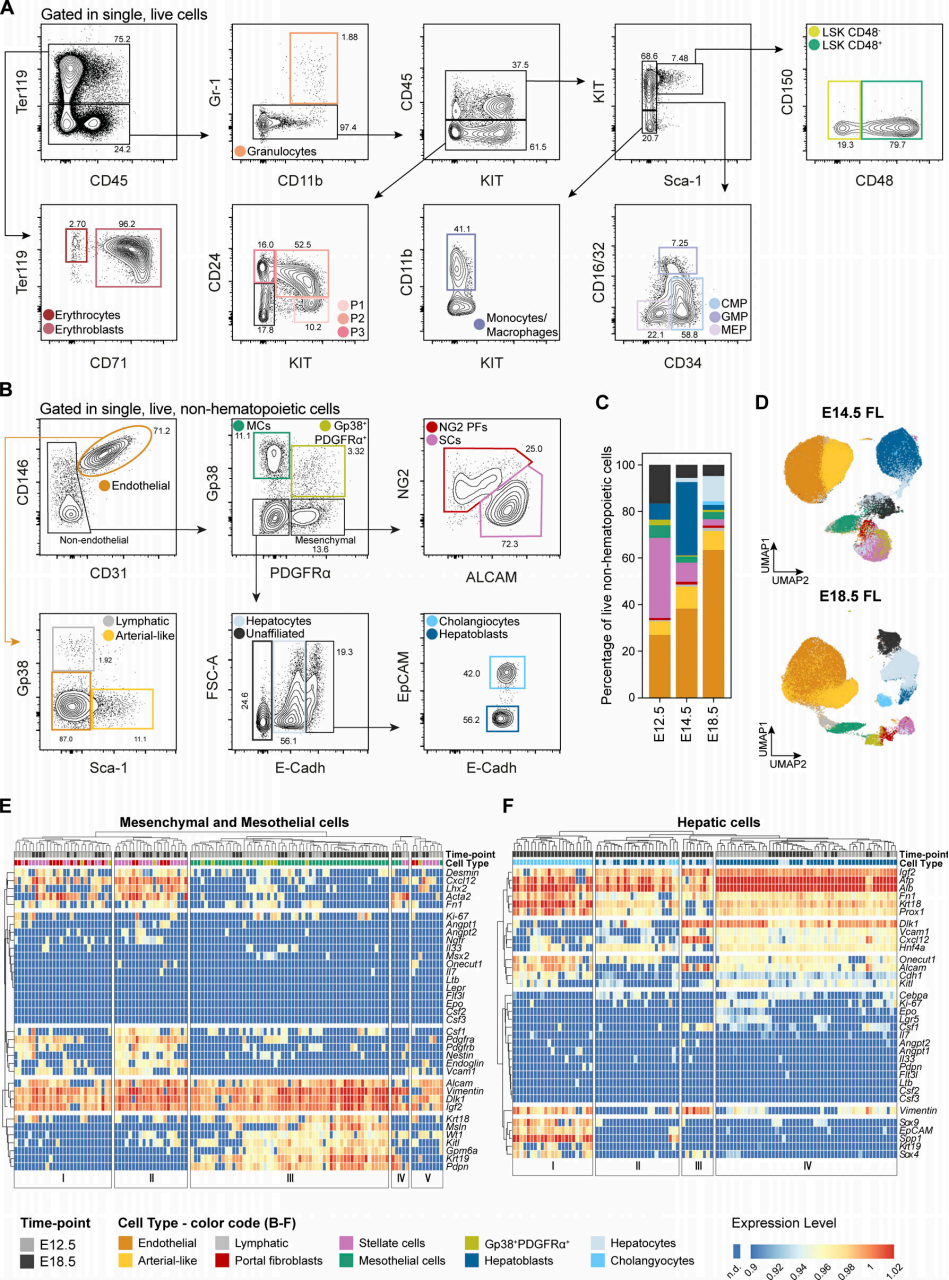
**Discrimination of the FL’s hematopoietic and stromal compartments. **Related to Fig. 1. **(A)** Gating strategy used for the analysis of the hematopoietic compartment of E12.5 FLs using the surface markers Ter119, CD45, CD71, Gr1, CD11b, KIT, Sca1, CD16/32, CD34, CD150, and CD48. Related to Fig. 1 B. **(B)** Gating strategy used for the analysis of Ter119^−^CD45^−^CD71^−^KIT^−^ FL cells using the surface markers CD146, CD31, Gp38, CD54, CD51, Thy1.2, PDGFRα, NG2, ALCAM, Sca1, E-Cadh, and EpCAM. Represented is the profile of E18.5. The same strategy was applied to all time points. **(C)** Distribution profile of live non-hematopoietic cells at E12.5, E14.5, and E18.5 as obtained from FC data. **(D)** UMAP analysis of FC data of Ter119^−^CD45^−^CD71^−^KIT^−^ cells from E14.5 and E18.5 FLs following the gating strategy represented in B. Color code as indicated in B. **(E and F)** Heatmap of single cell multiplex qPCR in sorted mesenchymal (E) and hepatic (F) cells of E12.5 and E18.5 FLs. Each column represents a single cell, and it is color-coded according to time point and cell type. Cells were sorted as indicated in B. Gene expression was normalized to *Actb* and *Gapdh*, and unsupervised hierarchical clustering was performed.

Using a set of surface markers (Table S1), we analyzed the hematopoietic compartment in the FL at E12.5 (Fig. 1 B and Fig. S1 A). Most of the cells belonged to the erythroid lineage (around 72% Ter119^+^CD71^+^ erythroblasts and 2% Ter119^+^CD71^−^ terminally differentiated erythrocytes). A heterogenous population of CD45^−^Ter119^−^ erythroid-committed progenitors (P1, P2, and P3), that we previously showed were derived from the YS (Soares-da-Silva et al., 2021), accounted for 10% of FL cells. CD45^+^ cells encompassing all other hematopoietic cells represented around 10%. The remaining fraction consisted of stromal cells, which are notably underrepresented following the isolation process.

We performed spectral FC analysis between E12.5 and E18.5 of the non-hematopoietic compartment (after depletion of Ter119^+^CD45^+^KIT^+^CD71^+^ cells), which represents <5% of total FL cells, with a panel of 12 surface markers (Table S1, Fig. 1 C, and Fig. S1, B–D). 10 cell types were identified and classified into four major populations: endothelial (CD31^+^CD146^+^), mesothelial (CD31^−^PDGFRα^−^Gp38^+^), mesenchymal (CD31^−^Gp38^−^PDGFRα^+^), and hepatic (CD31^−^Gp38^−^PDGFRα^−^E-Cadh^+^) cells (Fig. 1 C and Fig. S1 B).

Endothelial cells, the most represented population, expressed high levels of endothelial transcripts *Cdh5*, *Tek*, *Kdr*, *Endoglin*, *Nestin*, and *Efnb2*, and showed heterogeneous expression of *Ly6a* (*Sca-1*) and *Lepr* (Fig. 1 D). Minor subpopulations expressed Sca-1, classified as arterial-like (Kunisaki et al., 2013) (∼10%), or Gp38, classified as lymphatic (Breiteneder-Geleff et al., 1999; Saharinen et al., 2004) (∼1–2%, Fig. S1 B).

Within non-endothelial cells, Gp38 and PDGFRα further defined two populations: Gp38^+^ MCs, expressing specific mesothelial transcripts *Gpm6a*, *Msln*, *Wt1*, *Krt19*; and PDGFRα^+^ mesenchymal cells, expressing *Desmin*, *Nestin*, *Pdgfrb*, *Vcam1*, *Acta2*, *Lhx2*, *Ngfr* (Fig. 1 D and Fig. S1, B and E). We detected a population of CD31^−^Gp38^+^PDGFRα^+^ cells (mostly represented at E12.5) that expressed transcripts of both mesothelial and mesenchymal cells (Fig. S1 E, within cluster III), possibly representing cells undergoing EMT.

We previously showed that PDGFRα^+^ALCAM^+^NG2^−/low^ cells within the mesenchymal compartment represent maturing SCs that acquire a specific autofluorescent (AF) profile as they accumulate vitamin A granules and express the stellate transcripts *Lhx2* and *Desmin* (Peixoto et al., 2022) (Fig. S1 E, Clusters I and II). In contrast, PDGFRα^+^ALCAM^−/low^NG2^+^ cells, here referred to as PFs, did not exhibit a characteristic AF profile (Peixoto et al., 2022), they were barely detectable at E12.5 and increased in frequency as development progressed (Fig. 1 C and Fig. S1 D). Given that the NG2^+^ phenotype is typically associated with pericytes in different tissues, including the BM (Gao et al., 2018), we analyzed the presence of transcripts described for pericytes in other organs, such as *Endoglin*, *Nestin*, *Pdgfrb*, and *Vcam1*, in this population. We found that NG2^+^ cells and SCs exhibited similar transcriptional profiles based on the genes examined and clustered together (Fig. S1 E, Clusters I and II).

During the early stages of development (E12.5–E14.5), E-Cadh^+^ hepatic cells consisted primarily of hepatoblasts that exhibit an E-Cadh^high^EpCAM^−^ phenotype (Fig. 1 C and Fig. S1, B–D) and displayed high expression levels of hepatic transcripts, including *Dlk1*, *Afp*, *Alb*, *Vcam1*, *Alcam*, and *Hnf4a* (Fig. 1 D). A subpopulation of hepatoblasts co-expressed the progenitor marker *Lgr5* and the proliferation marker *Ki-67* (Fig. 1 D and Fig. S1 F, Cluster IV), potentially representing more immature progenitors (Prior et al., 2019). At E18.5, we observed a distinct population of E-Cadh^dim^ cells that we classified as hepatocytes based on their lower expression levels of *Dlk1*, *Afp*, *Igf2*, *Vcam1*, absence of *Lgr5* and *Vimentin*, and expression of the mature marker *Cebpa* (Fig. S1, B and F, Cluster II). Additionally, we observed a distinct population of E-Cadh^high^EpCAM^+^ biliary duct cells or cholangiocytes, which did not express *Dlk1*, upregulate *Krt18*, *Prox1*, *Onecut1*, and *Alcam*, and express the cholangiocyte transcripts *Sox4*, *Sox9*, *Krt19*, and *Spp1* (Fig. S1, B and F, Cluster I).

Interestingly, high levels of the *Dlk1* transcript, a marker that has been used to isolate hepatoblasts (Chou et al., 2013; Chou and Lodish, 2010; Tanaka et al., 2009; Tanimizu et al., 2003), were found not only in mesenchymal, as previously described (Khan et al., 2016), but also in MCs and Gp38^+^PDGFRα^+^ cells (Fig. S1, E and F), indicating that previous studies on DLK1-expressing FL cells concerned mixed populations.

### High-resolution landscapes of FL cellular components

**Figure 2.**
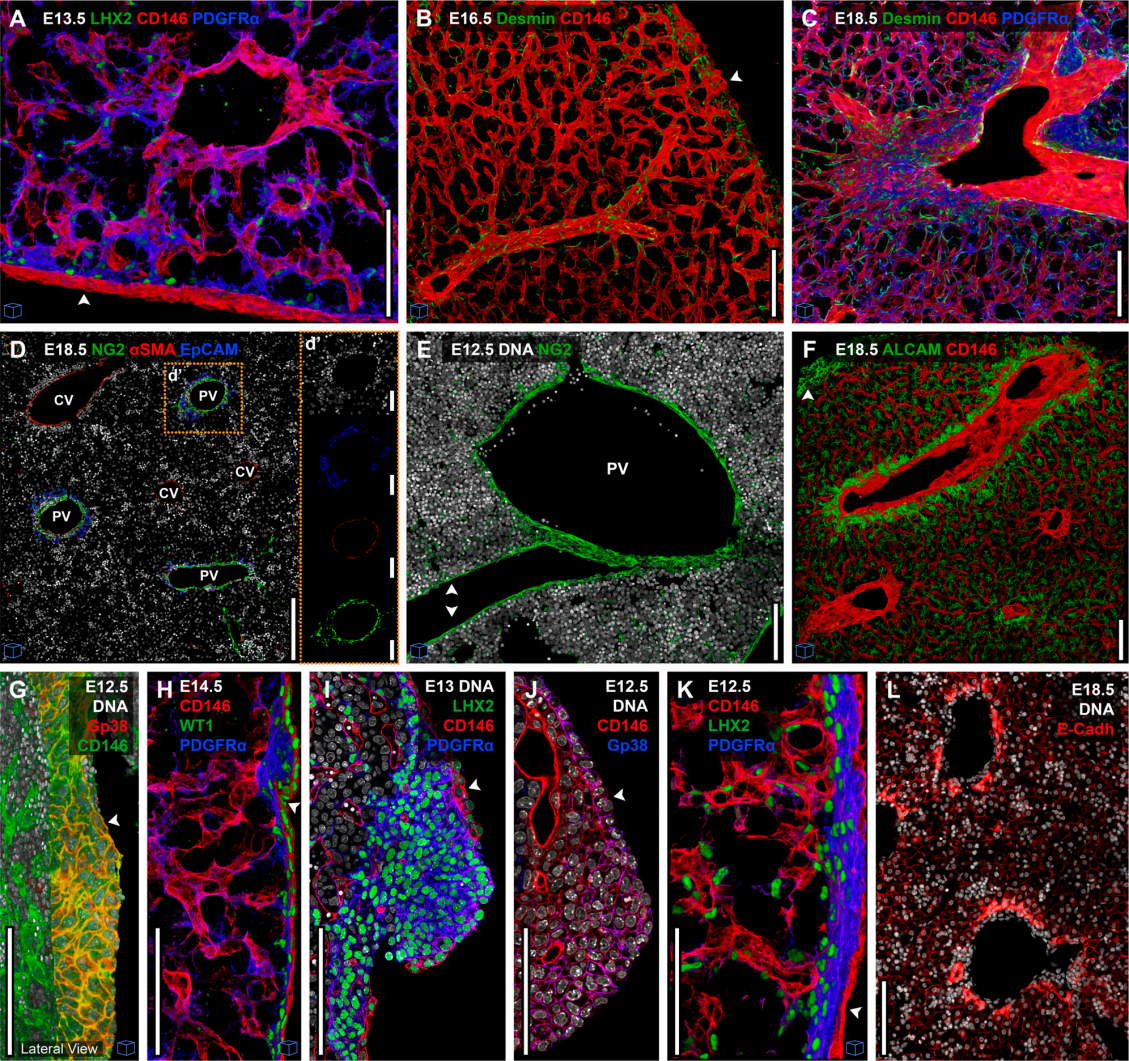
**Discrimination of the FL’s stromal compartments *in situ*. (A)** Representative 3D view (30 μm projection) IF of an E13.5 FL section with LHX2 (green), CD146 (red), and PDGFRα (blue). **(B)** Representative 3D view (50 μm projection) IF of an E16.5 FL section with Desmin (green) and CD146 (red). **(C)** Representative 3D view (57 μm projection) IF of an E18.5 FL section with Desmin (green), CD146 (red), and PDGFRα (blue). **(D)** Representative 3D view (10 μm) IF of an E18.5 FL section with DNA (white), NG2 (green), αSMA (red), and EpCAM (blue). **(d′)** The insert d′ shows the selected region with the channels separated. **(E)** Representative 3D view (20 μm projection) IF of an E12.5 FL section with DNA (white) and NG2 (green). **(F)** Representative 3D view (50 μm projection) IF of an E18.5 FL section with ALCAM (green) and CD146 (red). **(G)** Representative 3D view (50 μm projection) IF of an E12.5 FL section with DNA (white), Gp38 (red), and CD146 (green). **(H)** Representative 3D view (30 μm projection) IF of an E14.5 FL section with CD146 (red), WT1 (green), and PDGFRα (blue). **(I)** Single stack IF of an E13 FL section with DNA (white), LHX2 (green), CD146 (red), and PDGFRα (blue). **(J)** Single stack IF of an E12.5 FL section with DNA (white), CD146 (red), and Gp38 (blue). **(K)** 3D view (50 μm projection) IF of an E12.5 FL section with CD146 (red), LHX2 (green), and PDGFRα (blue). **(L)** Representative single stack IF of an E18.5 FL section with DNA (white) and E-Cadh (red). Arrowheads point to the mesothelium. Scale bars, 100 μm. CV, central vein; PV, portal vein. Images with cubes are 3D projections.

We located the tissue distribution of the stromal components (Fig. 1 E) with a three-color staining strategy: hepatoblasts, evenly scattered throughout the parenchyma, were recognized by their nuclear expression of HNF4α; and the intricate vascular and perivascular network of the FL and the mesothelial layer (clearly distinguished by its position) at the periphery could be mapped using CD146. Additionally, LHX2/PDGFRα/Desmin identified mesenchymal cells in the sub-mesothelium and perivascular areas (Fig. 1 E, Fig. 2, A–C; and Video 1). Whereas all types of vascular structures (i.e., sinusoids and main vessels) were surrounded by PDGFRα/LHX2/Desmin expression, only the main vessels (i.e., central and portal veins) expressed αSMA. Portal veins were surrounded by a particular type of mesenchymal cells, the NG2^+^ PFs, where at later stages bile ducts (EpCAM^+^) are formed (Fig. 2 D). Consistent with the FC data, NG2^+^ cells were scarce during the early stages and were only found surrounding the portal veins, namely the portal sinus (PS) in the center of the FL sections at E12.5 (Fig. 2 E). As development progressed, these cells became more abundant and their spatial distribution expanded significantly, aligning with the growth of the portal network (Fig. 2 D). Expression of ALCAM allowed the discrimination between mesenchymal subcompartments by FC, and we were able to assess its expression profile in the tissue. However, ALCAM was also expressed broadly across other stroma populations, namely hepatic, hindering clear visual discrimination between perivascular populations (Fig. S1, E and F; Fig. 2 F; and Video 2).

**Video 1.** **The intricate vascular and perivascular network of the E18.5 FL.** Video of a multi-stack or a rotative 3D view (50 μm) IF of an E18.5 FL section with Desmin (green), CD146 (red), and PDGFRα (blue). Scale bar, 100 μm. Playback speed is 24 frames per second.

**Video 2.** **ALCAM displays a widely distributed expression pattern across FL populations.** Video of a multi-stack and a rotative 3D view (50 μm) IF of an E18.5 FL section with ALCAM (green), CD146 (red), and NG2 (magenta). Scale bar, 100 μm. Playback speed is 24 frames per second.

MCs were easily recognized at the liver surface as a single epithelial layer of flattened CD146, Gp38, and WT1 expressing cells (Fig. 2, G and H). Underneath this layer, sub-MCs expressed PDGFRα, WT1, LHX2, and Desmin (Fig. 2, A, B, and H–L). NG2 was commonly observed to be expressed in MCs (Fig. 2 E, arrowheads), as previously reported (Ogawa et al., 2018).

Interestingly, we often found at E12.5, in some peripheral regions, a thick multilayer of sub-MCs and, at the tips of the liver lobes, an accumulation of cells that co-expressed PDGFRα, LHX2, CD146, and Gp38 (Fig. 2, I and J), also detected in FC (Fig. S1 B). At later stages, a monolayer of sub-MCs was predominant, but cell clusters at the tip of the lobes were still often detected.

At late gestation, the liver’s architecture is remarkably different from the early stages. We observed a shift from a densely packed organ, mainly constituted by hematopoietic cells, with an even distribution of hepatic cells (HNF4α^+^) (Fig. 1 E), to a structure with increased representation of hepatic cells, namely hepatocytes, which became juxtaposed, with a geometrical morphology (Fig. 2 L). As by FC, hepatocytes were identified as E-Cadh^dim^, while E-Cadh^high^ cells were only observed around the vessels (Fig. 2 L).

**Figure 3.**
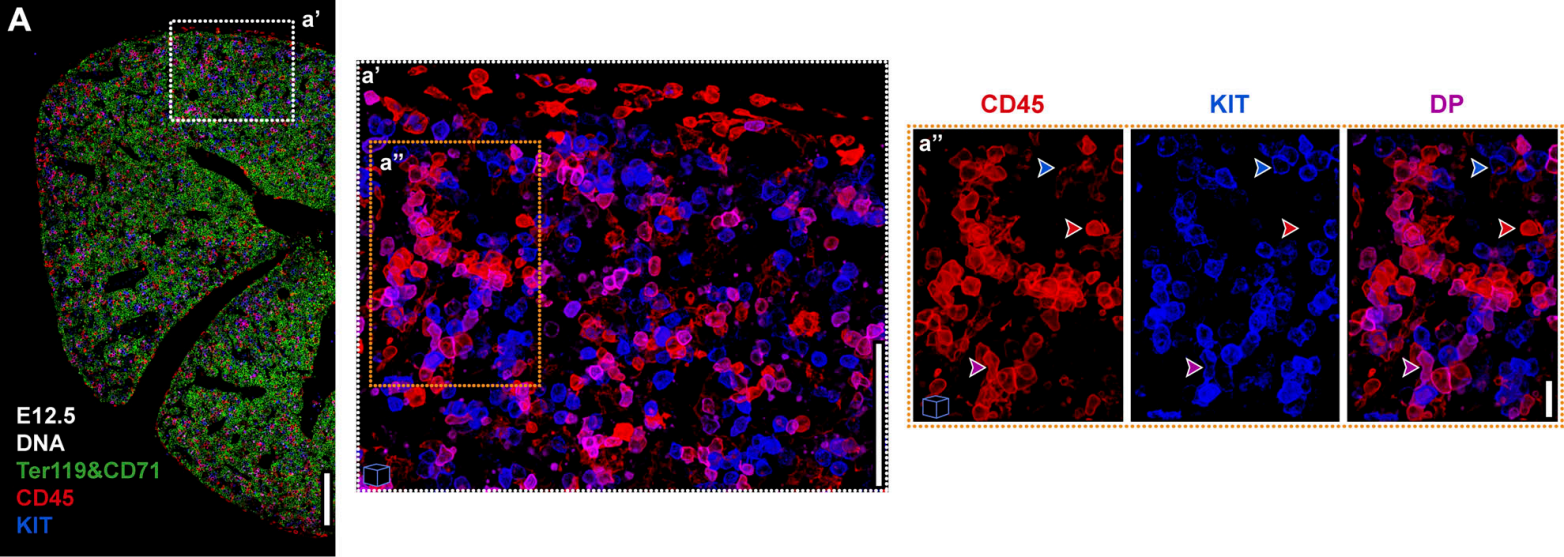
**Discrimination of CD45** ^**+**^ **, KIT** ^**+**^ **,** **or CD45** ^**+**^ **KIT** ^**+**^ **hematopoietic cells in cells in FL. (A)** Representative single-stack IF of an E12.5 FL section with DNA (white), Ter119, CD71 (green), CD45 (red), and KIT (blue). Scale bar, 200 μm. **(a′ and a″)** Inserts a′ and a″ represent enlarged 3D views (10 μm projections) of the selected regions. Scale bars: 100 μm (a′), 20 μm (a″). Blue (upper), red (middle), and purple (lower) arrowheads indicate an example of a KIT^+^, CD45^+^, or KIT^+^CD45^+^ (DP) cell, respectively. Images with cubes are 3D projections.

**Figure S2.**
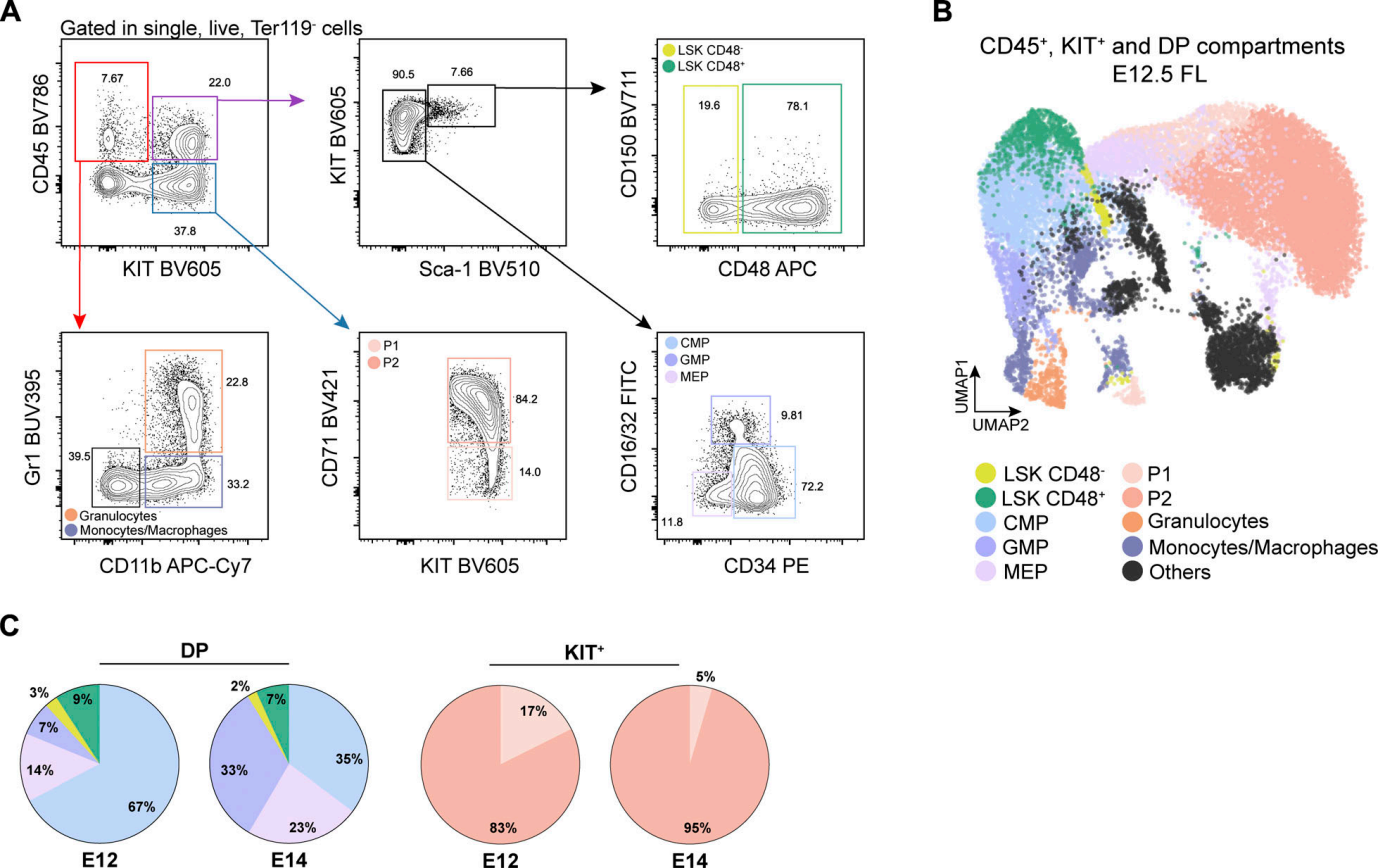
**Composition of the CD45** ^**+**^ **, DP, and KIT** ^**+**^ **compartments.** Related to Fig. 3. **(A and B)** Representative gating strategy (A) and UMAP analysis (B) showing the cell types that are included in CD45^+^ (CD45^+^KIT^−^), KIT^+^ (CD45^−^KIT^+^), and DP (CD45^+^KIT^+^) populations at E12.5. **(C)** Percentage of the progenitor cell types within DP cells (top) and KIT^+^ cells (bottom) at E12.5 (left) or E14.5 (right).

To visualize the majority of the hematopoietic compartment in FL sections, we combined the erythroid markers Ter119 and CD71, the pan-hematopoietic marker CD45, and the progenitor marker KIT (Fig. 3 and Fig. S2, A–C). The combination of CD45 and KIT staining allowed the identification of three major hematopoietic populations in the FL (Fig. 3 A). CD45^+^KIT^−^ cells, referred to as CD45^+^ cells, comprise mainly monocytes/macrophages and granulocytes at E12.5, whereas by E14.5 a small proportion of CD19 B lineage cells are also included in this subset, but no T or natural killer (NK) cells were present at this stage. CD45^−^KIT^+^ cells, referred to as KIT^+^ progenitors, comprise the YS-derived erythroid progenitors (P1 and P2) at E12.5–E14.5 (Soares-da-Silva et al., 2021); and CD45^+^KIT^+^, referred to as DP (double positive) progenitors, include a broad set of hematopoietic progenitors. The majority are LK cells (∼90%), with proportions of CLPs, CMPs, MEPs, and GMPs some of which may be of YS origin at this stage, which differ along development (Fig. S2 C). With this strategy, only a small proportion of DP (around 8%) are LSK CD48^−^ and LSK CD48^+^ cells.

### Pyxidis—An image analysis tool for tissue mapping

**Figure 4.**
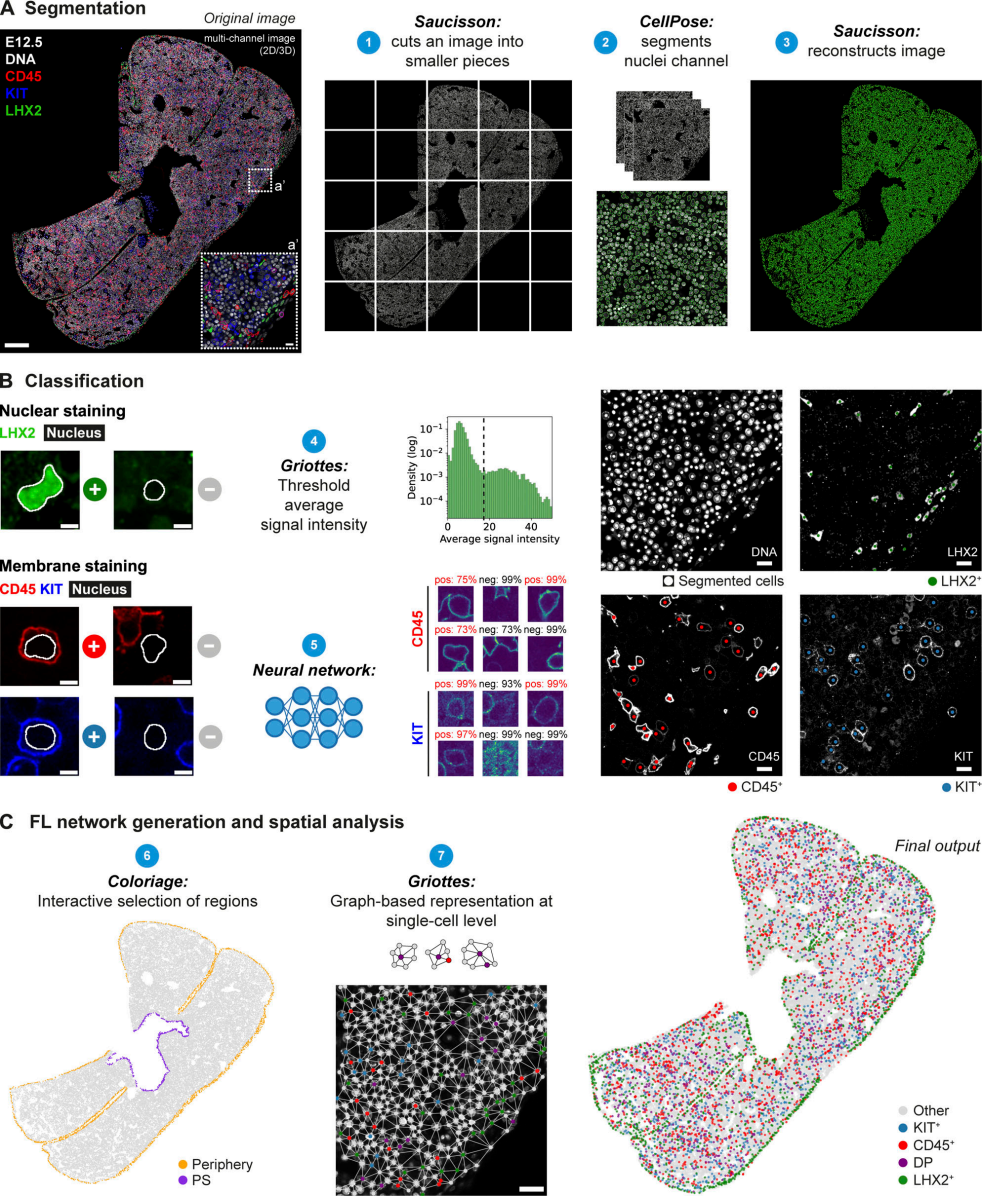
**Images analysis workflow. (A)** Segmentation pipeline. FL multichannel images are cut into small pieces using Saucisson (1), individual tiles are segmented based on the DAPI signal using CellPose (2), and then reassembled into the original shape (3). On the left, a representative single-stack IF of an E12.5 FL section with DAPI (white), LHX2 (green), KIT (blue), and CD45 (red) is shown to demonstrate the pipeline. Scale bar, 200 μm. **(a′)** The inset a′ represents an enlarged view of the selected region. Scale bar, 10 μm. **(B)** Cell type classification pipeline. In the case of nuclear staining (as for LHX2, green), cells are classified according to a threshold set on the average signal intensity inside the nuclei mask using Griottes (4). For membrane staining (e.g., for CD45/red, KIT/blue), cells are classified using a neural network (5). Individual tiles (80 × 80 or 120 × 120 pixels) are generated for each detected nucleus and centered around its geometric center, as represented on the left. Scale bars, 5 μm. Representative images of single cells classification and respective classification confidence (positive cells in red and negative in black) are shown in the middle panel. A representative example of a small tile post-classification is presented on the right, displaying segmented cells (in white) and cells positive for LHX2 (green), CD45 (red), and KIT (blue) (same region as shown in a′). Scale bars, 10 μm. **(C)** For FL network generation and spatial analysis, Coloriage is used to remove or highlight specific regions of the FL sections (6). Griottes is used to represent the cells in a dot plot and to plot the contacts between cells (7). The same region as represented in a′ is shown, with the classification color-coded in the dots, and the neighborhood interactions represented by the links. Scale bar, 10 μm.

To quantify the spatial distribution of different cell types, we built a multistep image analysis pipeline, which we named Pyxidis, to identify the phenotype and position of each cell within 2D and 3D (maximum ∼50 μm sections) images (Fig. 4). Pyxidis allows us to recreate FL sections in a graphical representation with preserved spatial positioning and cell identity in a compact format to perform multiple quantitative analyses: tissue composition, pattern of distribution of cells to specific regions or cell types, and neighborhood analysis. The pipeline integrates a series of published tools (Iandola et al., 2016, *Preprint*; Paszke et al., 2019, *Preprint*; Ronteix et al., 2022; Stringer et al., 2021) to perform three major steps: nucleus segmentation, cell classification, and network analysis (Fig. 4; see Materials and methods for detailed description).

The first step corresponded to the segmentation of the nuclei, based on the DAPI signal, using CellPose (Stringer et al., 2021). To optimize memory consumption, images were initially divided into smaller tiles (Fig. 4 A, 1), and segmentation was executed in parallel for each tile (Fig. 4 A, 2). Subsequently, the images were reconstructed to their original shape, with an additional channel of the nuclei’s mask (Fig. 4, A, 3).

**Figure S3.**
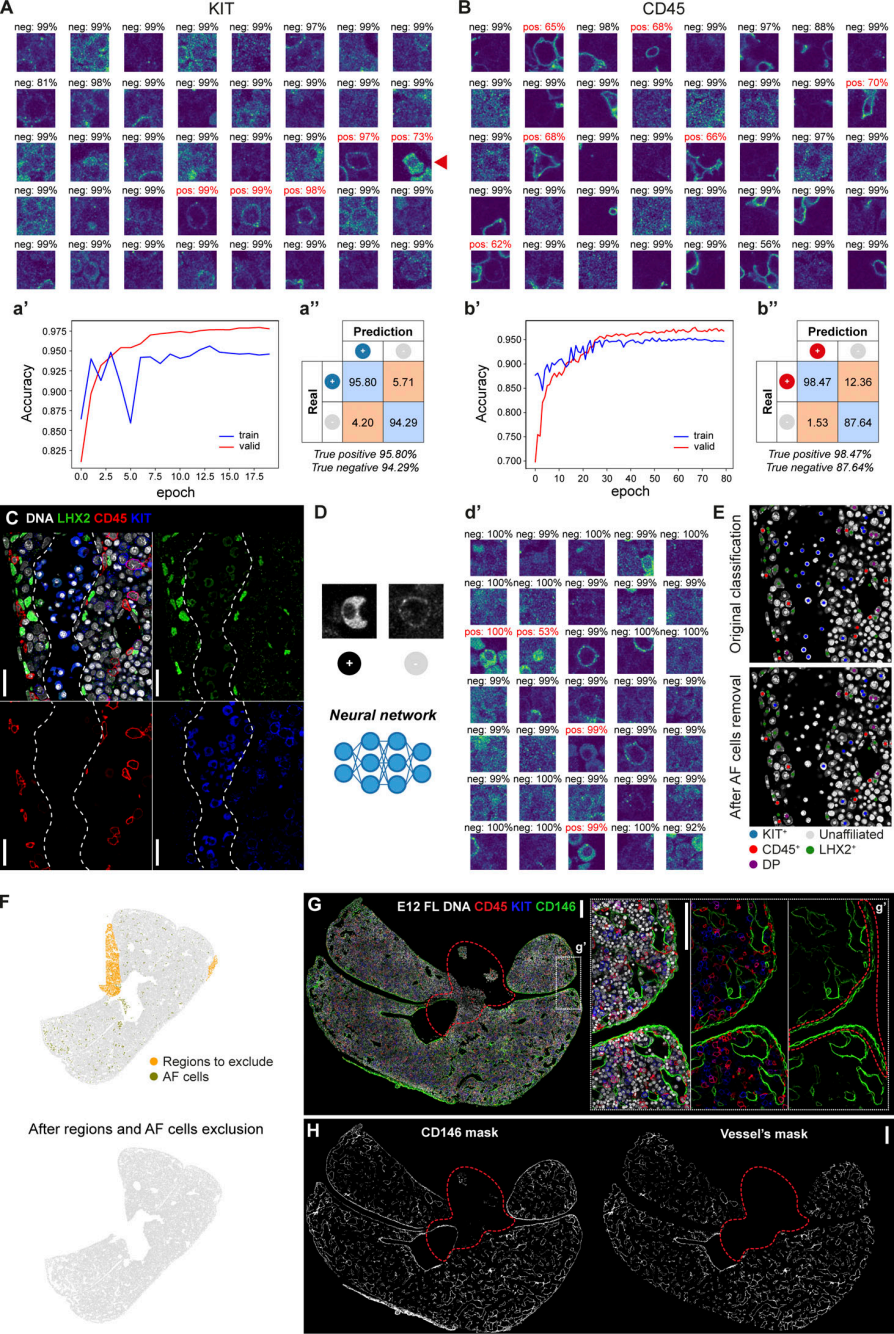
**Validation and quality control of the image analysis.** Related to Fig. 4. **(A and B)** Validation of KIT (A) and CD45 (B) classification. Representative images of single-cell classification and respective classification confidence (positive cells in red and negative in black). The red arrowhead indicates a false positive, corresponding to an AF cell. **(a′ and b′)** Accuracy of the classification during network training on training and validation datasets. **(a″ and b″)** Confusion matrix on the validation dataset. **(C)** Single stack IF of an E12.5 FL section with DNA (white), LHX2 (green), CD45 (red), and KIT (blue) to highlight a region where AF cells (nucleated erythrocytes) can be seen inside a blood vessel (represented by the dashed line). Scale bar, 25 μm. **(D)** AF cells are manually classified as positive and negative to train a neural network, so they can be later removed from the analysis. **(d′)** Representative images of single-cell classification and respective classification confidence (positive cells in red and negative in black). **(E)** DNA channel of the tile depicted in C overlaid with the classification before (top) and after (bottom) removing AF cells. **(F)** FL section (same input image as in Fig. 3) before and after removing regions manually selected using Coloriage, and AF cells, highlighted on the left panel at orange and olive green, respectively. **(G and H)** Vessel’s network segmentation. **(G)** Representative single stack IF of an E12.5 FL section with DNA (white), CD45 (red), KIT (blue), and CD146 (green). Scale bar, 200 μm. The dashed region represents a region to exclude from the analysis using the tool Coloriage due to lower staining quality in the CD146 channel. **(g′)** Insert g′ shows an enlarged view of the selected region. Scale bar, 100 μm. The dashed region represents a manual selection (in FIJI) corresponding to the mesothelial layer (CD146^+^) to exclude from the analysis. **(H)** Mask of CD146 signal obtained in FIJI (left) and mask of the vessels’ network obtained by manual exclusion of the CD146 signal in the mesothelial layer (right).

**Figure 5.**
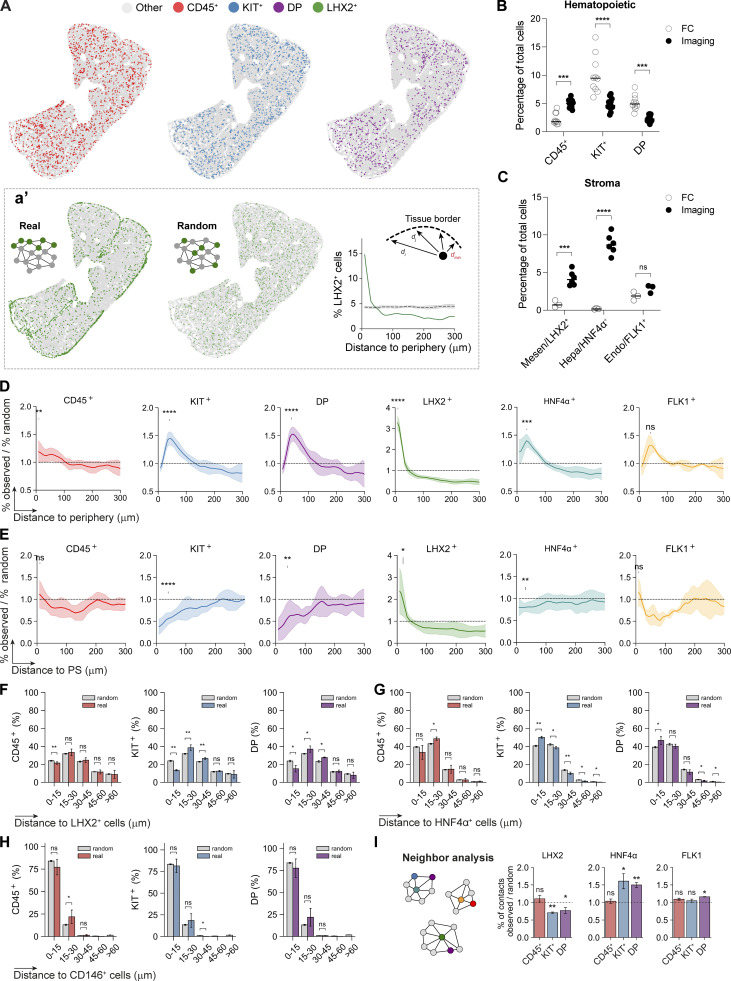
**YS and IE progenitors accumulate near the periphery at E12.5 and are overall preferentially in contact with hepatoblasts, but not with stellate or endothelial cells. (A)** Dot plot representation of CD45^+^ (red), KIT^+^ (blue), DP (purple), and LHX2^+^ (green) cells distribution of an E12.5 FL representative section. **(a′)** Experimentally observed (“real”) versus random LHX2^+^ cells distribution (green), and their corresponding distance to the periphery profile (green curve) cells in comparison with a random distribution (dashed line, average from 10 random distributions generated, with standard deviation in gray). The minimum Euclidean distance between each cell in the network and the cells marked as “periphery” is computed and the minimal distance for each data point is considered. **(B)** Percentage of hematopoietic cells recovered and analyzed by FC (*n* = 9) versus quantified by imaging of FL sections (*n* = 13, 521,423 total cells analyzed). **(C)** Percentage of stromal cells recovered and analyzed by FC (*n* = 3) versus quantified by imaging of FL sections (LHX2, *n* = 6, 608,788 total cells analyzed, HNF4α, *n* = 6, 605,984 total cells analyzed, and FLK1, *n* = 3, 97,602 total cells analyzed). Endo (CD146^+^CD31^+^), Hepa (CD31^−^Gp38^−^PDGFRα^−^EpCAM^−^E-Cadh^+^), and Mesen (CD31^−^Gp38^−^PDGFRα^+^). **(B and C)** Statistical analysis was calculated using two-way ANOVA, followed by Šídák’s multiple comparisons test. **(D)** Distance to the periphery profile (normalized to random) of CD45^+^ (red, *n* = 13, 521,423 total cells analyzed), KIT^+^ (blue, *n* = 13, 521,423 total cells analyzed), DP (purple, *n* = 13, 521,423 total cells analyzed), LHX2^+^ (green; *n* = 6, 608,788 total cells analyzed), HNF4α^+^ (cyan, *n* = 6, 605,984 total cells analyzed) and FLK1^+^ (orange, *n* = 2, 97,602 total cells analyzed) of E12.5 FL sections. Curves indicate average ± SD. Statistical analysis was calculated at the peak of each curve using Welch's *t* test, between observed and random profiles. **(E)** Distance to the PS profile (normalized to random) of CD45^+^ (red, *n* = 11, 488,919 total cells analyzed), KIT^+^ (blue, *n* = 11, 488,919 total cells analyzed), DP (purple, *n* = 11, 488,919 total cells analyzed), LHX2^+^ (green; *n* = 6, 608,788 total cells analyzed), HNF4α^+^ (cyan, *n* = 6, 605,984 total cells analyzed) and FLK1^+^ (orange, *n* = 2, 97,602 total cells analyzed) cells of E12.5 FL sections. Curves indicate average ± SD. Statistical analysis was calculated at 10–40 μm using Welch's *t* test, between observed and random profiles. **(F–H)** Spatial distribution of CD45^+^ (red), KIT^+^ (blue), and DP (purple) cells relative to LHX2^+^ (*n* = 3, 90.706 total cells analyzed, F), HNF4α^+^ (*n* = 3, 87,902 total cells analyzed, G) and CD146^+^ (*n* = 3, 60,050 total cells analyzed, H) cells. Plots show the distribution of each hematopoietic population after 10 rounds of randomization in gray (random). **(I)** Percentage of contacts (normalized to random) of CD45^+^ (red), KIT^+^ (blue), and DP (purple) cells with LHX2^+^ (*n* = 3, 90,706 total cells analyzed), HNF4α^+^ (*n* = 3, 87,902 total cells analyzed), and FLK1^+^ (*n* = 2, 97,602 total cells analyzed) stromal cells of E12.5 FLs. Bar plots indicate average ± SD. Statistical analysis was calculated using the Mann–Whitney test. *n* indicates the number of independent biological samples analyzed. *, P <0.05; **, P < 0.01; ***, P < 0.001; ****, P < 0.0001; ns, not significant.

In the second step, cells were classified according to their phenotype based on either nuclear or membrane staining (Fig. 4 B). For nuclear markers, such as LHX2, a fluorescence threshold was determined for each image, based on the histogram of signal intensities inside the nuclei mask (Fig. 4 B, 4). For membrane markers, such as CD45 and KIT, cells were classified using a deep learning-based neural network (Fig. 4 B, 5) (Iandola et al., 2016, *Preprint*; Paszke et al., 2019, *Preprint*), with accuracies larger than 95% (Fig. S3, A and B). The quality of the segmentation and classification steps was controlled by overlaying original images and dot plots of the resulting classifications for each image and visually testing the agreement (Fig. 4 B, right panels; Video 3).

**Video 3.** **Visual confirmation of the segmentation and classification using Napari.** Representative demonstration of the use of the graphical interface Napari to visually inspect the segmentation and classification. For the demonstration, the same FL section represented in Fig. 3 A was used. It corresponds to a single stack, stained with DNA (white), LHX2 (green), CD45 (red), and KIT (blue). Playback speed is 24 frames per second.

**Figure 6.**
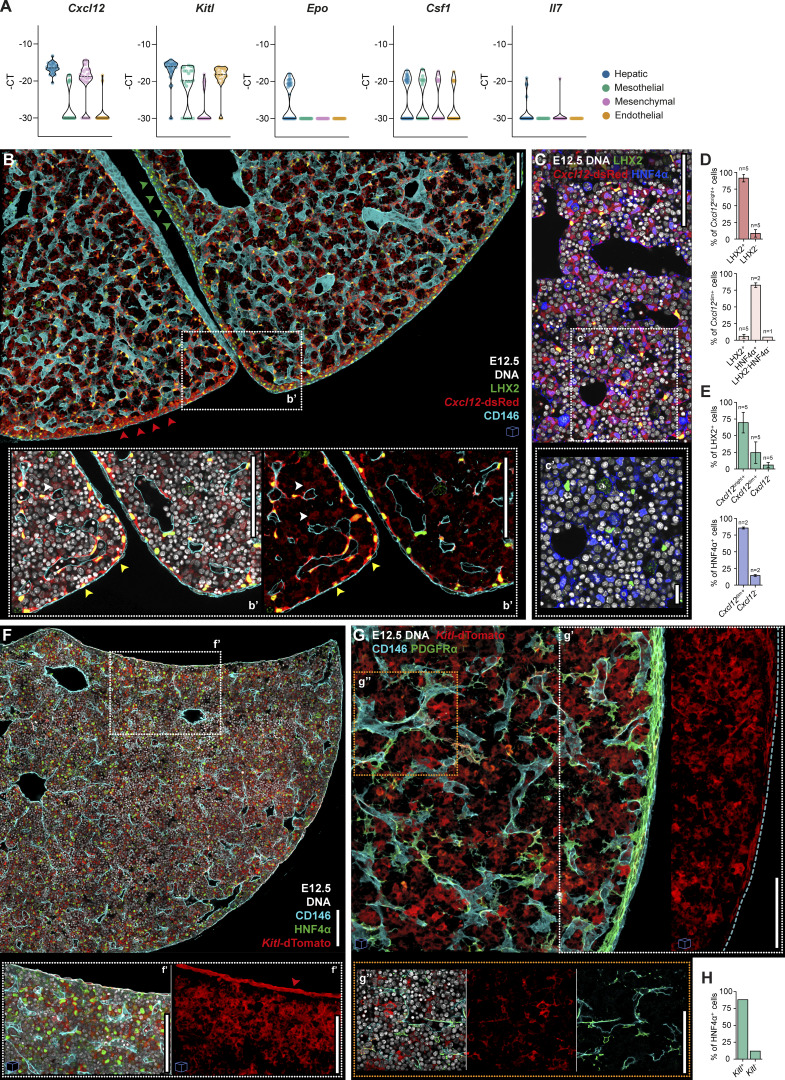
**Supportive hematopoietic cytokines are produced by multiple stromal populations. (A)** Violin plots of single-cell gene expression analysis of hematopoietic cytokines in hepatic (CD31^−^Gp38^−^PDGFRα^−^EpCAM^−^E-Cadh^+^, blue), mesothelial (CD31^−^PDGFRα^−^Gp38^+^, green), mesenchymal (CD31^−^Gp38^−^PDGFRα^+^, pink) and endothelial (CD31^+^CD146^+^, orange) cells at E12.5. Each dot represents a cell. **(B)** Representative 3D view (25 μm projection) IF of an E12.5 FL section (*Cxcl12*-dsRed mice) with LHX2 (green), *Cxcl12*-dsRed (red) and CD146 (cyan). Red arrowheads point to a sub-mesothelium region with *Cxcl12*-dsRed^bright^ cells, while green arrowheads indicate a region with *Cxcl12*-dsRed^dim^ cells. **(b′)** Insert b′ shows an enlarged single-stack view of the selected region. DNA is shown in white. Yellow arrowheads indicate cells with high expression of *Cxcl12*, co-staining with LHX2. White arrowheads indicate cells with low expression of *Cxcl12*, LHX2^−^. Scale bars, 100 μm. **(C)** Representative single-stack IF of an E12.5 FL section (*Cxcl12*-dsRed mice) with DNA (white), LHX2 (green), *Cxcl12*-dsRed (red), and HNF4α (blue). Scale bar, 100 μm. **(c′)** Insert c′ shows an enlarged single-stack view of the selected region. Scale bar, 20 μm. **(D)** Quantification of LHX2^+^ and HNF4α^+^ cells within *Cxcl12*-dsRed^bright+^ and *Cxcl12*-dsRed^dim+^ cells. **(E)** Quantification *Cxcl12*-dsRed cells with distinct signal intensities in LHX2^+^ and HNF4α^+^ stromal cells. **(D and E)** In total, six images from independent biological samples were analyzed, corresponding to a total of 241,647 cells. The specific *n* for each condition is indicated above the bars. **(F)** Representative single-stack IF of an E12.5 FL section (*Kitl*-dTomato mice) with DNA (white), HNF4α (green), *Kitl*-dTomato (red), and CD146 (cyan). **(f′)** Insert f′ shows an enlarged 3D view (20 μm projection) of the selected region. Scale bar, 100 μm. Red arrowhead indicates a region of the mesothelium with high expression of *Kitl*-tdTomato. **(G)** Representative 3D view (15 μm projection) IF of an E12.5 FL section (*Kitl*-dTomato mice) with PDGFRα (green), *Kitl*-dTomato (red), and CD146 (cyan). **(g′ and g″)** Inserts g′ and g″ show an enlarged 3D view (15 μm projection) or a single stack of the selected regions, respectively. DNA is shown in white (g″). The dashed line in g′ represents the mesothelium with no expression of *Kitl*-tdTomato. Scale bar, 100 μm. **(H)** Quantification of *Kitl*-dTomato^+^ cells in HNF4α^+^ cells. HNF4α^+^ classified cells from a single image (3D image, *n* = 1, 16,000 total cells) were selected and *Kitl*-dTomato^+^ cells were manually classified. *n* indicates the number of independent biological samples analyzed. Images with cubes are 3D projections.

We found nucleated erythrocytes in sinusoids exhibiting a cytoplasmic AF signal in one or more channels (Fig. S3 C) that could compromise the accuracy of the classification of membrane markers (e.g., Fig. S3 A, red arrowhead). To address this issue, we employed the same neural network approach to identify and subsequently exclude cells detected as AF^+^ from the analysis (Fig. S3, D–F). Parts of the tissue (e.g., disrupted lobes, other organs adjacent to the FL, or regions with lower quality) were also excluded from the analysis. A specific interactive tool named Coloriage was created to graphically select regions in the segmented image. This tool was used to select undesired regions (Fig. S3 F) or to mark structures (e.g., the liver’s periphery, vessels, individual lobes) for downstream analysis (Fig. 4 C, 6).

After occasional manual corrections performed in regions of lower staining quality and AF^+^ cells exclusion, the frequency of false positives and false negatives, which ranged from 0% to 7%, was determined for each image by manual confirmation of 400 randomly selected individual cells (Table S2).

After the segmentation, the location of each cell was identified by considering the geometric center of its respective nucleus, presuming that the distance from this center to the cell’s membrane remained consistently small, from 4 to 10 μm. This assumption held well for hematopoietic cell types (except Mks and some macrophages), and mesenchymal cells and hepatoblasts. Since endothelial membrane markers are challenging to attribute to a single nucleus using the neural network, we used the *Flk1*-GFP reporter mice to identify and quantify these cells, using the threshold method for FLK1 classification. However, because these cells have elongated shapes (Fig. 2 A), we complemented this analysis by segmenting the vasculature based on the CD146 signal (Fig. S3, G and H). Since CD146 also stains MCs, we manually selected the mesothelial layer (Fig. S3, G g′, dashed line) and excluded it to generate a mask specifically representing the CD146 signal within the vessels (Fig. S3 H). These two complementary approaches allowed an accurate assessment of the distribution of hematopoietic cells in relation to endothelial cells.

**Figure 7.**
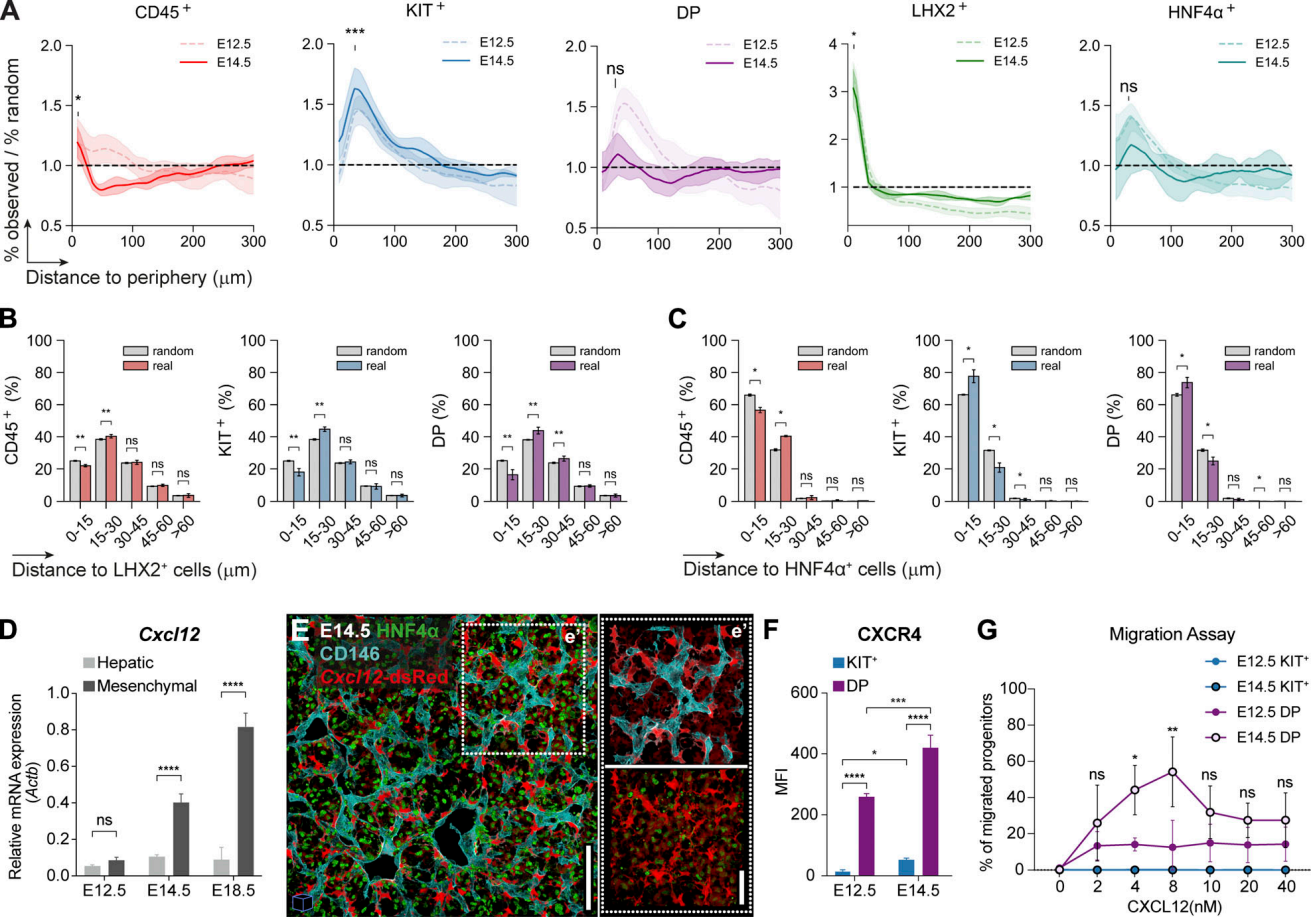
**At E14.5, opposite to YS-, IE-derived progenitors display a homogeneous distribution throughout the parenchyma in response to *Cxcl12* levels. (A)** Distance to the periphery profile (normalized to random) of CD45^+^ (red, *n* = 6, 557,370 total cells analyzed), KIT^+^ (blue, *n* = 6, 557,370 total cells analyzed), DP (purple, *n* = 6, 557,370 total cells analyzed), LHX2^+^ (green, *n* = 3, 320,278 total cells analyzed), and HNF4α^+^ (*n* = 3, 237,092 total cells analyzed) cells of E14.5 FLs. E12.5 data from Fig. 4 B is overlaid for comparison. Curves indicate average ± SD. Statistical analysis was calculated at the peak of each population using Welch's *t* test, between observed and random profiles at E14.5. **(B and C)** Spatial distribution of CD45^+^ (red), KIT^+^ (blue), DP (purple) cells relative to LHX2^+^ (*n* = 3, 320,278 total cells analyzed, B) and HNF4α^+^ (*n* = 3, 237,092 total cells analyzed, C). Statistical analysis was calculated using the Mann–Whitney test. **(D)** qRT-PCR analysis of the expression levels of the hematopoietic chemokine *Cxcl12* in hepatic (CD31^−^Gp38^−^PDGFRα^−^EpCAM^−^E-Cadh^+/high^, light gray), and mesenchymal (CD31^−^Gp38^−^PDGFRα^+^, dark gray) cells in E12.5, E14.5 and E18.5 FL cells (*n* = 3). **(E)** Representative 3D view (25 μm projection) IF of an E14.5 FL section (*Cxcl12*-dsRed mice) with HNF4α (green), *Cxcl12*-dsRed (red), and CD146 (cyan). Scale bar, 100 μm. **(e′)** Insert e′ shows an enlarged view of the selected region. Scale bar, 50 μm. **(F)** Mean fluorescence intensity (MFI) of CXCR4 in KIT^+^ (blue), and DP (purple) cells in E12.5, E14.5 FLs (*n* = 3). **(G)** Transwell migration assay of DP and KIT^+^ progenitors of E12.5 and E14.5 FL in response to the chemokine CXCL12a. Data are presented as the percentage of input progenitors that migrate to the bottom chamber (*n* = 3). **(D–G)** Statistical analysis was calculated using two-way ANOVA, followed by Šídák’s multiple comparisons test. *n* indicates the number of independent biological samples analyzed. *, P < 0.05; **, P < 0.01; ***, P < 0.001; ****, P < 0.0001; ns, not significant.

Pyxidis replaces the pixel/voxel-scale description with a cell-scale representation of the complete image, either in 2D or in 3D. In this representation, each cell is identified by its position and is characterized by the expression of the nuclear or membrane markers (Fig. 4 C, final output). This representation is stored as a connected graph using the Griottes package (Ronteix et al., 2022), which identifies the neighbors of each cell (Fig. 4 C, 7). This graph enables a robust statistical analysis of the spatiotemporal organization of the different cell types during FL development and quantitatively describes cell–cell interactions.

### Spatial organization and interactions between stromal and hematopoietic compartments at E12.5

To localize the FL hematopoietic and stromal components, we used CD45 and KIT for hematopoietic cells, in combination with each of the stromal cell markers LHX2 (mesenchymal) (Fig. 5 A), HNF4α (hepatic), or FLK1/CD146 (endothelial). As MCs correspond to the outer layer of the liver, they were manually selected using the tool Coloriage and classified as “periphery” (Fig. 4 C, 6). Pairwise comparisons between the frequencies of each population evaluated by FC and imaging revealed marked differences (Fig. 5, B and C). Stromal cells were significantly underestimated (5–10-fold) in FC, which indicates the difficulties in obtaining single-cell suspensions of these populations. The most affected subset was the hepatoblasts and the less affected the endothelial cells. In contrast, KIT^+^ and DP cells did not differ more than twofold with lower frequencies detected in imaging compared to FC. However, because stromal cells and also CD45^+^ cells (myeloid/macrophages) were severely underrepresented in FC, we assume that the twofold lower frequency of the former reflects a compensatory effect and that they are faithfully detected in both strategies.

The graph-based description of FL sections provided a global view of the spatial distribution of the cell types (Fig. 5 A). To quantify these distributions, we calculated the relative percentage of each cell type at a given distance to the liver’s periphery and the PS (central vessel structure), and normalized by the percentage of each cell type in an average random distribution (%observed/%random) (Fig. 5, D and E). Random distributions of cells were computed by shuffling the position of the different cell types while preserving the structure of the tissue and the frequency of each cell type (Fig. 5, A a′). We observed a significant yet small enrichment of CD45^+^ cells close to the periphery (∼15% increase of CD45^+^ cells at this location), while not differing significantly from the random distribution in the parenchyma (Fig. 5 D). Interestingly, KIT^+^ and DP progenitor cells exhibited a pronounced enrichment at 50 μm from the liver’s periphery (50% increase) but lower frequency in the vicinity of the PS. As visually evident, LHX2^+^ mesenchymal cells accumulated in the sub-mesothelium region (sub-MCs), were enriched adjacent to the PS (PFs), and were homogeneously scattered throughout the parenchyma (SCs) (Fig. 5 A). HNF4α^+^ hepatoblasts and FLK1^+^ also displayed a higher frequency close to the periphery (Fig. 5 D). However, the dataset with FLK1 is not significant precluding robust conclusions.

To evaluate the spatial relationship between hematopoietic and stromal cells, we measured the distance of each hematopoietic population (CD45^+^, KIT^+^, and DP) to the nearest stromal cell (LHX2^+^, HNF4α^+^) or the vascular network (CD146^+^) (Fig. 5, F–H). The distribution of CD45^+^ cells appeared random when compared with HNF4α^+^ cells and vessels and exhibited a (small) lower frequency of cells in close/direct contact with LHX2^+^ cells. In contrast, KIT^+^ and DP progenitors showed a pronounced decrease in frequency within the 0–15 μm range and an increase at the 15–45 μm range away from LHX2^+^ cells (Fig. 5 F). Conversely, the analysis of the proximity to HNF4α^+^ cells demonstrated the opposite trend, with enrichment of cells closer to hepatoblasts than in locations further away, in comparison with random distributions (Fig. 5 G). Although the majority of cells of any hematopoietic type (around 75%) were close to a vascular structure, this distribution was not different from the random scenario (Fig. 5 H), reflecting the high abundance of vessels within the FL (Fig. 1, E e′). In agreement, analysis of cell–cell contacts showed that CD45^+^ cells did not display a discernible tendency for preferential neighbors. In contrast, KIT^+^ and DP progenitors exhibited a higher frequency of contacts with hepatoblasts and lower with SCs compared with a random distribution (Fig. 5 I).

Altogether, these results identify the peripheral region of the FL as a privileged site for hematopoietic progenitors at E12.5 and an overall preferential association with hepatoblasts and avoidance tendency for direct contact with SCs.

### Supportive hematopoietic cytokines are produced by distinct stromal populations and accumulate at the mesothelial and sub-mesothelial regions

Hematopoietic cytokines are crucial in the regulation of hematopoiesis as they play a fundamental role in promoting survival, proliferation, and differentiation of progenitors into various hematopoietic cell types. Cytokine expression was analyzed in single cells of the four major stromal populations, revealing hepatoblasts as predominant cytokine producers at E12.5 (Fig. 6 A and Fig. S1, E and F). These cells expressed high levels of *Cxcl12* and *Kitl* and were the only producers of *Epo*. A subfraction expressed intermediate levels of *Csf1*, and a few cells expressed low levels of *Il7*. Another two major producers of *Kitl* were mesothelial and endothelial cells that were also heterogeneous regarding *Csf1* expression. *Cxcl12* was also produced at high levels by virtually all mesenchymal cells (Fig. 6 A and Fig. S1 E, Clusters I and II). Of note, all hepatoblasts that co-expressed *Cxcl12* and *Kitl*, as well as also *Epo* and *Il7*, clustered together (Fig. S1 F, Cluster IV) and appeared to be the most immature cells because they co-expressed the progenitor marker *Lgr5* and *Ki67*. In contrast, co-expression of *Csf1* and *Cxcl12* was detected in different cells within cluster IV and in cluster II (E18.5) (Fig. S1 F). In the hierarchical clustering analysis, *Csf1-expressing* mesenchymal cells were mostly represented in Cluster II, which differed from Cluster I by the expression of the stellate markers *Lhx2* and *Ngfr* (Fig. S1 E). Interestingly, *Igf2*, encoding an important growth factor during development (Sélénou et al., 2022) was expressed at very high levels by nearly all FL stromal cells analyzed (Fig. S1, E and F).

To characterize the tissue distribution of the two most expressed cytokines, CXCL12 and KITL, we analyzed FLs from reporter *Cxcl12*-dsRed (Ding and Morrison, 2013) and *Kitl*-dTomato mice (Buono et al., 2016) (Fig. 6, B–H; and Videos 4 and 5). We identified two distinct levels of *Cxcl12* intensity *in situ*. The cells with high levels of *Cxcl12*-dsRed co-expressed LHX2 and were predominantly located in the sub-mesothelial and perivascular regions (Fig. 6 B). The expression of *Cxcl12* in the sub-mesothelial region was found to be heterogeneous, with some regions displaying an accumulation of cells expressing bright levels of *Cxcl12*-dsRed (Fig. 6 B, red arrowheads), while others appear to express low-to-no levels (Fig. 6 B, green arrowheads). Cells with a dull expression of *Cxcl12* were found in the parenchyma and co-expressed HNF4α (Fig. 6, B b′, white arrowheads, Fig. 6 C). As detected by gene expression analysis, nearly all mesenchymal cells and hepatoblasts expressed *Cxcl12* (Fig. 6, A, D, and E). Cells expressing *Kitl*-tdTomato displayed two main distribution patterns. They lined the FL, in a mesothelial position, co-expressing CD146, and were scattered throughout the parenchyma in HNF4α^+^ cells (Fig. 6 F). Of note, mesothelial expression of *Kitl* was found to be heterogeneous, with some regions exhibiting low or no signal (Fig. 6, F f′, red arrowhead, Fig. 6, G g′, dashed line). Virtually all HNF4α^+^ cells were *Kitl*-tdTomato^+^ (Fig. 6, A, F, and H). Despite endothelial cells displaying a consistent expression of *Kitl* in the single-cell analysis, due to their flat morphology, the specific cellular source of *Kitl* in the vicinity of endothelial cells remains unclear *in situ*, as it often coincides with PDGFRα staining (Fig. 6, G g″ and Video 5).

**Video 4.** **Pattern of expression of *Cxcl12* in *Cxcl12*-dsRed reporter mice at E12.5.** Video of a multi-stack and a rotative 3D view (25 μm) IF of an E12.5 FL section with LHX2 (green), Cxcl12-dsRed (red), and CD146 (cyan). Scale bar, 100 μm. Playback speed is 24 frames per second.

**Video 5.** **Pattern of expression of Kitl in Kitl-tdTomato reporter mice at E12.5.** Video of a multi-stack and a rotative 3D view (20 μm) IF of an E12.5 FL section with LHX2/HNF4α (green), Cxcl12-dsRed (red), and CD146 (cyan). Scale bar, 100 μm. Playback speed is 24 frames per second.

**Figure S4.**
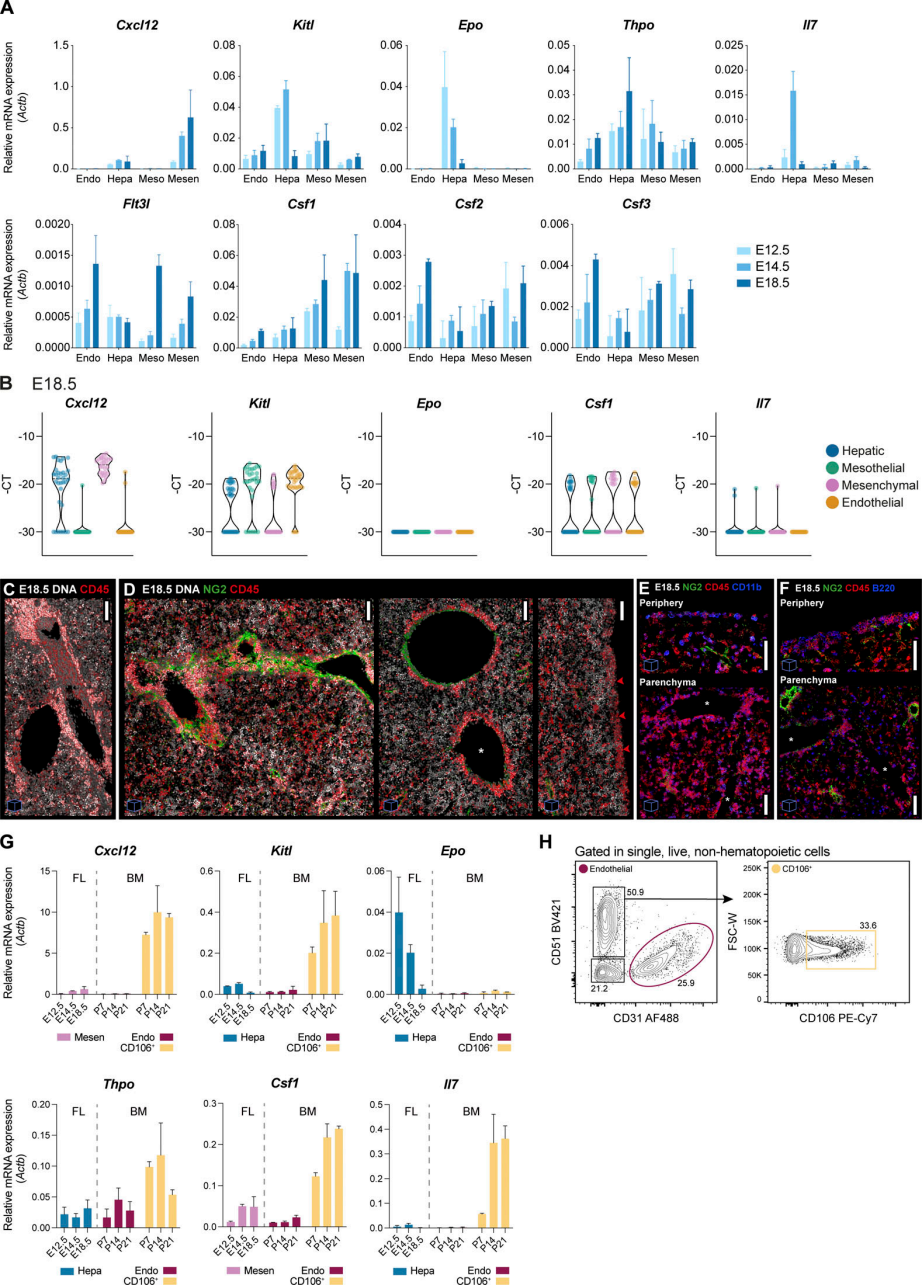
**Cytokines’ expression along development.** Related to Fig. 6. **(A)** qRT-PCR analysis of the expression levels of hematopoietic cytokines in endothelial (Endo, CD31^+^CD146^+^), hepatic (Hepa, CD31^−^Gp38^−^PDGFRα^−^EpCAM^−^E-Cadh^high^), mesothelial (Meso, CD31^−^PDGFRα^−^Gp38^+^), and mesenchymal (Mesen, CD31^−^Gp38^−^PDGFRα^+^) cells in E12.5 (light blue), E14.5 (blue), and E18.5 (dark blue) FL cells (*n* = 3). **(B)** Violin plots of single-cell gene expression analysis of hematopoietic cytokines in hepatic (blue), mesothelial (green), mesenchymal (pink), and endothelial (orange) cells at E18.5. Each dot represents a cell. **(C)** 3D view (45 μm projection) IF of an E18.5 FL section with DNA (white) and CD45 (red). Scale bar, 100 μm. **(D)** 3D view IF of three E18.5 FL sections (30, 25, and 20 μm projections, from left to right) with DNA (white), NG2 (green) and CD45 (red). Scale bars, 100 μm. **(E)** 3D view (20 μm projection) IF covering the peripheral region of the liver (upper panel) or the parenchymal region (lower panel) with NG2 (green), CD45 (red), and CD11b (blue). Scale bar, 50 μm. **(F)** 3D view (20 μm projection) IF covering the peripheral region of the liver (upper panel) or the parenchymal region (lower panel) with NG2 (green), CD45 (red), and B220 (blue). Scale bar, 50 μm. **(D–F)** * represent central veins. The red arrows in D point to the mesothelial layer. **(G)** qRT-PCR analysis of the expression levels of hematopoietic cytokines in mesenchymal (Mesen, pink) or hepatic (Hepa, blue) cells of E12.5, E14.5, and E18.5 FL and endothelial (dark red) or CD51^+^CD106^+^ (yellow) cells of P7, P14, and P21 BM. For each cytokine, only the FL population with the highest expression levels is shown (*n* = 3). **(H)** Gating strategy used for the analysis of the stroma of P7, P14, and P21 BM using the surface markers CD51, CD31, and CD106. Representative profile of a P7 BM. The same strategy was applied to all time points. *n* indicates the number of independent biological samples analyzed. All bar plots indicate average ± SD.

To gain a comprehensive understanding of the cytokine expression dynamics in the FL during development, we conducted an analysis of key hematopoietic cytokines in all stromal populations from E12.5 to E18.5 (Fig. S4, A and B). Our findings showed that mesenchymal cells were the major producers of *Cxcl12* after E12.5, and levels of this chemokine increased at later stages. Conversely, *Kitl*, *Epo*, and *Il7* were predominantly expressed by hepatoblasts and exhibited a decrease in expression at E18.5. Of note, we observed that, unlike earlier stages, fewer hepatoblasts express *Cxcl12* and *Kitl* at E18.5 (Fig. S4 B). *Tpo*, primarily expressed by hepatoblasts, showed increased expression levels at later stages, as reported before (Lee et al., 2022).

The cytokines critical for myeloid (*Csf1*) and lymphoid (*Flt3*) differentiation show increased expression with development in endothelial cells, mesenchymal cells, and MCs while remaining nearly constant in hepatoblasts. In line with this, we observed frequent accumulations of CD45^+^ mature cells (both myeloid and lymphoid cells) in E18.5 FLs around the major vessels (either NG2^−^ vessels or NG2^+^ portal vessels) and in the vicinity of the mesothelial regions (Fig. S4, C and D).

To understand how the FL cytokine levels compare with other hematopoietic microenvironments, we additionally assessed cytokine expression from two major stromal populations in the BM: endothelial cells (CD31^+^) and a population enriched in mesenchymal cells (CD51^+^CD106^+^), putatively CAR cells, the major cytokine producer in the BM (Baryawno et al., 2019) (Fig. S4, G and H). Because BM development is only initiated at the late stages of gestation and undergoes major changes in the first weeks after birth, we analyzed multiple time points, from postnatal days 7, 14, and 21. Except for *Epo*, which is expressed in the kidney from late gestation to adulthood (Suzuki et al., 2011), and not in the BM, all other cytokines were expressed at significantly higher levels by the CD106^+^ BM cells (Fig. S4, G and H). These observations suggest that the FL stromal populations are less proficient in cytokine production, which might result in a comparatively lower cytokine-rich environment in the FL, as compared with the BM.

### At later stages, IE-derived but not YS-derived progenitors display a homogeneous distribution throughout the liver

**Figure S5.**
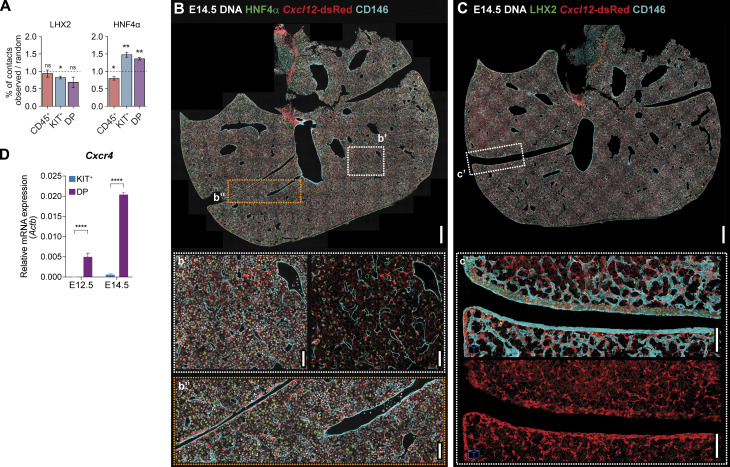
**Image analysis of E14.5 FL sections.** Related to Fig. 7. **(A)** Percentage of contacts (normalized to random) of CD45^+^ (red), KIT^+^ (blue), and DP (purple) cells with LHX2^+^ (*n* = 3, 320,278 total cells analyzed) and HNF4α^+^ (*n* = 3, 237,092 total cells analyzed) stromal cells of E14.5 FLs. Statistical analysis was calculated using Welch's *t* test. **(B)** Representative single-stack IF of an E14.5 FL section (*Cxcl12*-dsRed mice) with DNA (white), HNF4α (green), *Cxcl12*-dsRed (red), and CD146 (cyan). Scale bar, 200 μm. **(b′ and b″)** Inserts b′ and b″ show enlarged views of the selected regions. Scale bars, 50 μm. **(C)** Representative single-stack IF of an E14.5 FL section (*Cxcl12*-dsRed mice) with DNA (white), LHX2 (green), *Cxcl12*-dsRed (red), and CD146 (cyan). Scale bar, 200 μm. **(c′)** Insert c′ shows an enlarged 3D view (25 μm projection) of the selected region. Scale bars, 50 μm. **(D)** qRT-PCR analysis of the expression levels of the CXCL12 receptor (*Cxcr4*) in KIT^+^ (blue), and DP (purple) cells in E12.5, E14.5 FLs (*n* = 3). Statistical analysis was calculated using two-way ANOVA, followed by Šídák’s multiple comparisons test. All bar plots indicate average ± SD. *n* indicates the number of independent biological samples analyzed. *, P < 0.05, **, P < 0.01; ****, P < 0.0001.

Given that the FL is a major site of hematopoiesis during development, we investigated the distribution of hematopoietic cells at subsequent developmental stages. At E14.5, we found that KIT^+^ progenitors maintained their accumulation close to the periphery while DP progenitors were evenly distributed in the parenchyma (Fig. 7 A). CD45^+^ cells were found at a lower frequency, at 50 μm distance from the periphery, and maintained a mild increase in the vicinity of the mesothelium. LHX2^+^ cells exhibited a similar concentration in the sub-MC region as at E12.5 but with an increased frequency of cells throughout the parenchyma. The concentration of HNF4α^+^ cells near the periphery was less evident and not statistically significant (Fig. 7 A). The distance of the three hematopoietic subsets to LHX2^+^ and HNF4α^+^ cells was similar to E12.5 but differed in the representation of hepatoblasts (Fig. 7, B and C). In a random distribution, >60% of the cells were close to hepatoblasts (∼25% more than at E12.5), but still at a lower frequency (∼10% lower) than that observed for KIT^+^ and DP progenitors. In contrast, the frequency of CD45^+^ cells in contact with hepatoblasts was lower than expected if randomly distributed. Furthermore, our analysis of cell–cell contacts demonstrated that, similar to earlier time points, KIT^+^ and DP hematopoietic progenitors showed a preferential interaction with hepatoblasts, but not with SCs (Fig. S5 A).

We aimed to investigate the mechanisms underlying the changes in spatial distribution of DP progenitors by analyzing the expression of CXCL12 and its receptor CXCR4 involved in hematopoietic cell migration. During development, *Cxcl12* is upregulated in mesenchymal cells, reaching an eightfold increase between E12.5 and E18.5, while it was maintained constant at lower levels in hepatoblasts (Fig. 7 D). At E14.5, the pattern of expression in the *Cxcl12*-dsRed was similar to E12.5, with clear discrimination between perivascular *Cxcl12*-dsRed^bright^ and HNF4α^+^*Cxcl12*-dsRed^dim^ (Fig. 7 E and Fig. S5, B and C). CXCR4 was expressed by DP at E12.5 and increased levels at E14.5 (Fig. 7 F), also reflected in mRNA levels (Fig. S5 D). By contrast, KIT^+^ cells were devoid of CXCR4 expression. To confirm differences in migration responsiveness to CXCL12 of the two types of progenitors, we conducted a dual-chamber migration assay. As expected, only DP progenitors migrated in response to CXCL12, and the proportion of migrating cells was more pronounced at E14.5 (Fig. 7 G).

We hypothesize that E14.5 DP progenitors migrate from the periphery into the parenchyma in response to changes in CXCL12 expression. We propose that the perivascular upregulation of CXCL12 creates a gradient that attracts DP progenitors, dispersing the cells that were accumulated around 50 μm from the periphery. Because the MCs and sub-MCs produce *Kitl* and *Cxcl12*, which might act as synergetic chemokines (Christensen et al., 2004), but also differentiation factors, like *Flt3l* and *Csf1* (Fig. S4 A), they might confer to the liver’s periphery a unique microenvironment, attracting hematopoietic progenitors at late stages (E18.5) and inducing their differentiation, leading to the accumulation of mature cells in this location at later stages (Fig. S4, C–F).

### Unlike DP, LSK enriched for CD48^−^ cells, do not preferentially neighbor hepatoblasts

**Figure 8.**
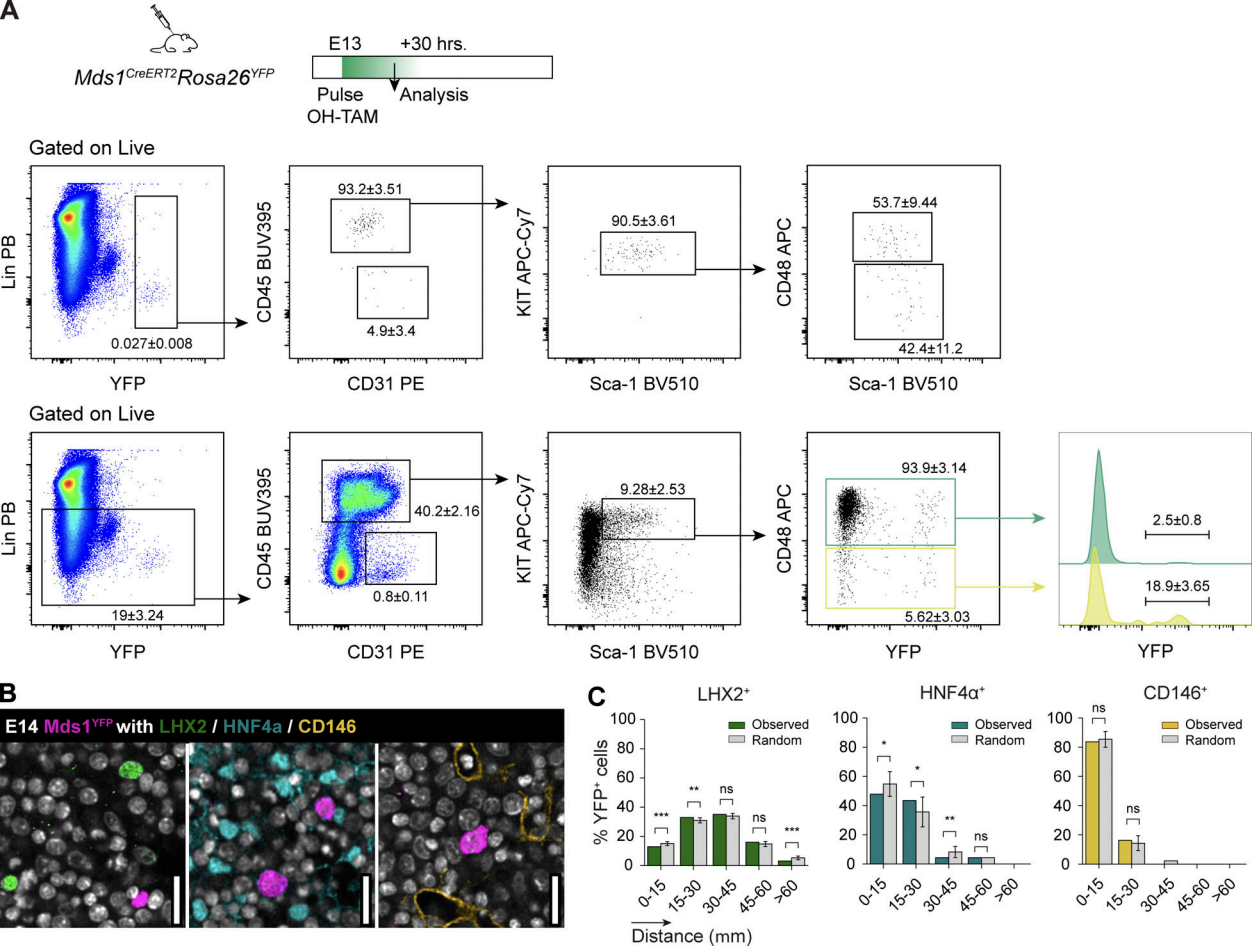
**CD48** ^**−**^ **LSK are not in close vicinity to hepatoblasts. (A)** Experimental strategy to analyze LSK enriched for CD48^−^ cells from Mds1^CreERT2^Rosa26^YFP^ mice and gating strategy to analyze the phenotype of YFP^+^ cells. Frequencies ± SD of three independent FL analyzed from two independent experiments. **(B)** Representative single-stack IF of an E14.5 FL section (Mds1^CreERT2^YFP mice) with DNA (white), YFP (magenta), and LHX2 (green), HNF4α (cyan), or CD146 (orange). Scale bar, 30 μm. **(C)** Spatial distribution of YFP^+^ cells relative to LHX2^+^ (84 YFP^+^ cells analyzed), HNF4α^+^ (44 YFP^+^ cells analyzed), and CD146^+^ (58 YFP^+^ cells analyzed) cells. Statistical analysis was calculated using a *t* test one sample. *, P < 0.05; **, P < 0.01; ***, P < 0.001; ns, not significant.

As our staining strategy comprises a very heterogenous pool of hematopoietic progenitors (Fig. S2), we used the Mds1^CreERT2^ YFP mouse line to define a population highly enriched for adult type HSC (Fig. 8 A) and characterize their interaction with stromal cells (Fig. 8, B and C). YFP^+^CD45^+^ cells represented over 90% LSK cells, ∼40% being CD48^−^ cells (Fig. 8 A). Due to the limited labeling efficiency in this experimental setting, a comprehensive spatial analysis of this population was not feasible. Instead, we focused on their distribution relative to three main stromal populations (LHX2^+^, HNF4α^+^, and CD146^+^ cells). Our analysis indicated that these rare cells do not preferentially neighbor LHX2^+^ or CD146^+^ cells, nor do they show a preference for direct contact to HNF4α^+^ cells, as total DP (Fig. 8, B and C).

### 3D spheroids of stromal and hematopoietic cells in culture reveal the relevance of hepatoblasts and KITL in LSK expansion

**Figure 9.**
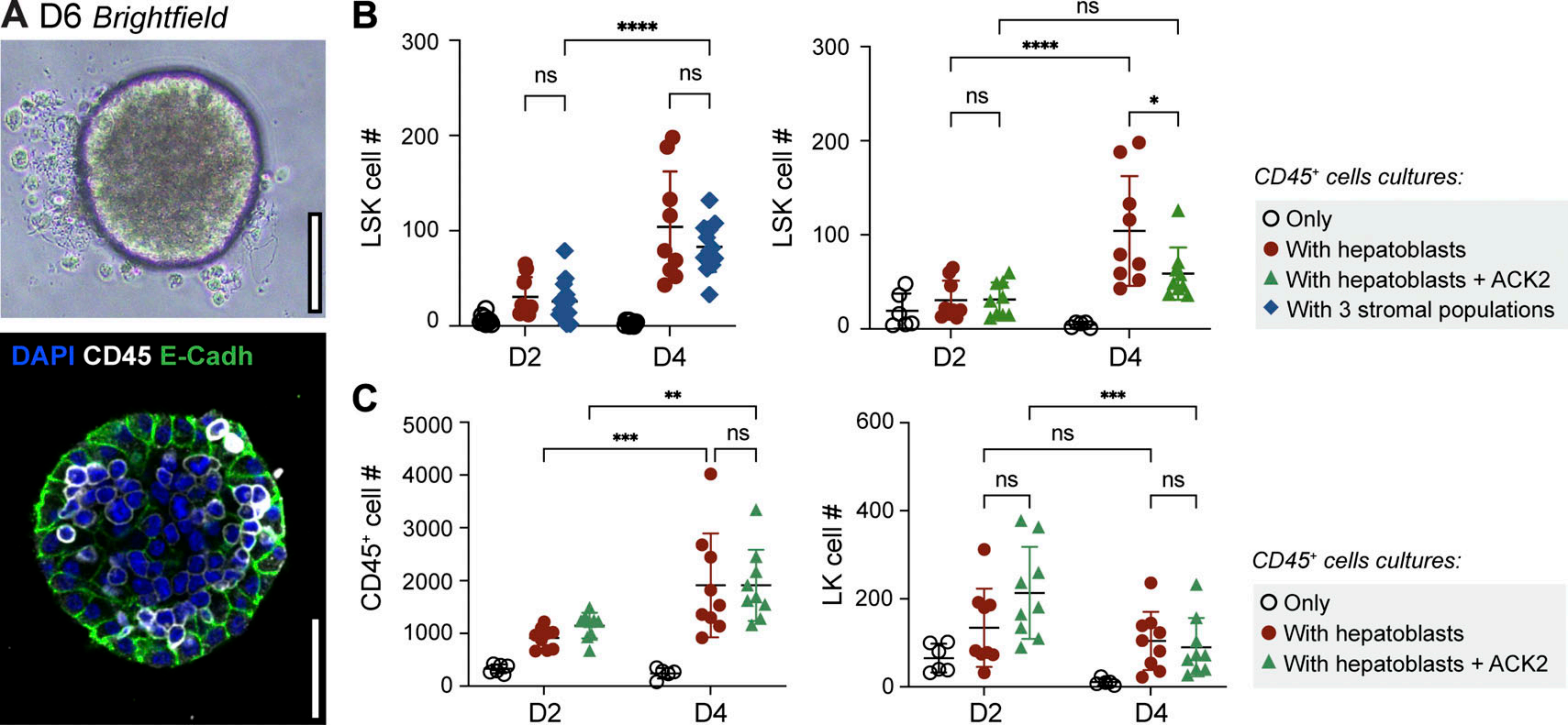
**Hepatoblasts and KITL alone are sufficient to expand FL LSK. (A)** Image of a spheroid at day 6 (D6) of culture (upper image, brightfield) and representative single-stack IF of a D6 spheroid of a co-culture of E14.5 hepatoblasts and CD45^+^ FL cells; DNA (blue), E-Cadh (green), and CD45 (white). **(B)** Numbers of LSK cells recovered from spheroids after 2 (D2) and 4 (D4) days of culture. Cultures with three populations: hepatoblasts (CD31^−^Gp38^−^PDGFRα^−^EpCAM^−^E-Cadh^+^ cells), mesenchymal cells (CD31^−^Gp38^−^PDGFRα^+^ cells), and MCs (CD31^−^PDGFRα^−^Gp38^+^). ACK2 antibody was added on D0 of CD45^+^ cells co-cultured with hepatoblasts. **(C)** Numbers of CD45^+^ cells and Lin^−^Kit^+^Sca-1^−^ (LK) cells isolated from individual spheroids (*n* = 3). All bar plots indicate average ± SD. *n* indicates the number of independent biological samples analyzed. Statistical analysis was calculated using two-way ANOVA, followed by Tukey’s multiple comparison test. *, P < 0.05; **, P < 0.01; ***, P < 0.001; ****, P < 0.0001; ns, not significant.

To understand the differential role of stromal cells in hematopoietic development, we devised a spheroid 3D assay that contained either hepatoblasts alone or a combination of hepatoblasts, mesenchymal cells, and MCs at relative proportions similar to those found *in vivo* (Fig. 9). We co-cultured CD45^+^ FL cells in either condition and found that, within 2 days, LSK increased by fourfold irrespective of being co-cultured with hepatoblasts or with the three stromal subsets, indicating that hepatoblasts alone could support LSK expansion (Fig. 9 B, left panel). Adding ACK2, a KIT-specific antibody that competes with KITL binding (Nishikawa et al., 1991), to the spheroids inhibited the expansion of LSK (Fig. 9 B, right panel). Moreover, although the total number of CD45^+^ cells was not affected, we observed a reduction in LK, between D2 and D4 of culture in the presence of ACK2 (Fig. 9 C) indicating that KITL has a critical role in the expansion of hematopoietic progenitors in FL.

## Discussion

### Methodology

We analyzed FL hematopoiesis throughout development, focusing on the E12.5–E14.5 window, where HSCs are found to increase sharply (Ema and Nakauchi, 2000). Although the degree of HSC expansion in the FL is still under debate (Ganuza et al., 2022, 2017; Soares-da-Silva et al., 2023), the need to dissect the major hematopoietic site during development persists. We confirmed the affiliation of FC-resolved cell types by single-cell multiplexed gene expression and were able to identify their location *in situ* using multiple markers. Overall, up to E14.5, a set of three markers was sufficient to simultaneously identify the majority of FL stromal cell types by imaging: LHX2/Desmin/PDGFRα for the mesenchymal compartment, HNF4α for hepatic cells, and CD146 for the vascular and perivascular network, and additionally, the mesothelial layer. Despite the availability of robust histological atlases of the FL during development (Crawford et al., 2010; Swartley et al., 2016), which have been instrumental in understanding tissue architecture and remodeling, there is still a scarcity of IF studies that identify both the hepatic and hematopoietic systems simultaneously.

Toward this goal, we built a dataset of FL sections that show the spatial distribution of the stromal and hematopoietic populations with a single-cell resolution. A dedicated image analysis pipeline (Pyxidis) was developed to enable a quantitative characterization of the cellular organization in these tissues. The tool provided a method to interrogate each cell in the image and assign a cell type to it from the fluorescent signal while preserving spatial and neighborhood information. This allowed multiple downstream quantitative analyses: evaluation of tissue composition, distribution patterns, and neighbor analysis. A total of 1.8 × 10^6^ cells from 51 tissue slices were thus analyzed, which yielded robust statistics about the spatial cell organization. The reduction of the data size that results from the Pyxidis pipeline enables these single-cell data to be shared easily.

Compared with FC, this method not only preserved spatial information but also captured certain cell types that were underestimated due to the dissociation procedures of the FC, particularly mesenchymal cells and hepatoblasts. CD45^+^ cells that comprise tissue-resident macrophages, which are easily recognized by their irregular morphology, were also underestimated. Altogether, the increase in the frequency of stromal and CD45^+^ cells in imaging analysis resulted in a compensatory moderate decrease (twofold) in the frequency of KIT^+^ and DP cells. In this regard, the ability to perform a visual quality control of the cell type identification from randomly selected images (Table S2) confirmed the improved robustness of Pyxidis compared with classical FC.

### Cytokine profiling

Cytokines/chemokines are considered key players in the development of the hematopoietic system. We analyzed hematopoietic cytokine production by the different stromal subsets by transcription analysis and using reporter mouse lines. We found that multiple cell types produce the same cytokine/chemokine albeit at different levels. This explains why depletion of these cytokines in specific cell types, which is a widely employed approach to functionally assess stromal populations, resulted in an absence of discrete hematopoietic phenotypes in the FL (reviewed by Crane et al. [2017], Gao et al. [2018]). Using a 3D spheroid culture system, we showed that *in vitro* expansion of the LSK progenitor compartment can be driven by hepatoblasts and is mediated by KITL. Our results also indicate that cytokines expression (KITL, EPO, IL7) decreases at later developmental stages in hepatoblasts. By then only mesenchymal and endothelial cells express KITL bringing a rationale for why targeting deletion of this cytokine to these two cell types resulted in a severe hematopoietic deficiency, found only in the perinatal stage (Lee et al., 2021).

PFs Nestin^+^NG2^+^ were identified as major players in the FL hematopoietic niche, producing *Cxcl12*, *Kitl*, *Igf2*, and *Angptl3* (Khan et al., 2016). We showed that *Cxcl12* is also expressed by all mesenchymal cells, including PFs, SCs, and sub-MCs. NG2 is additionally expressed in a subset of MCs that also express *Kitl*, *Csf1*, and *Tpo* and that we identify as a, yet unrecognized, regulator in FL hematopoietic microenvironment. Therefore, NG2^+^ cell depletion might result in unpredictable consequences for the integrity of the mesothelial compartment and for the organ architecture which could contribute to the decrease in HSC observed by Khan et al. (2016).

When comparing the levels of transcripts in sorted FL and BM stromal populations, we observed a big disparity between the populations of the two niches. We previously found that YS-derived erythroid progenitors outcompete HSC-derived progenitors for access to low concentrations of EPO present in FL because they have a lower threshold response to this factor (Soares-da-Silva et al., 2021). Moreover, the low concentration of IL-7 in the FL allows B1 B cell selection for autoreactive specificities (Wong et al., 2019). Although we cannot directly infer the local protein availability of these factors, a relatively low cytokine environment and a higher threshold for cytokine response in HSC could explain their limited contribution to fetal hematopoiesis (Yokomizo et al., 2022).

### Spatiotemporal cell distribution

Using Pyxidis, we consistently noticed that KIT^+^ and DP progenitors are enriched at 50 μm from the periphery at E12.5. At this stage, some regions of the liver feature a dense layer of sub-MCs that exhibit a high expression of *Cxcl12*, possibly contributing to elevated levels of this chemokine in the vicinity of the liver’s periphery. Two sources of KITL were also enriched in this region: some MCs adjacent to the sub-mesothelial layer, and hepatoblasts, also enriched 50 μm from the periphery. As MCs secrete hepatic growth factors (PTN, HGH, EGF, MDK) (Inagaki et al., 2015; Onitsuka et al., 2010), we propose that the periphery of the liver is a privileged site for hepatoblasts proliferation. As both hematopoietic progenitors were shown to be preferentially in contact with hepatoblasts, the existence of a multicellular circuit being established near the periphery could explain our observations. The hepatoblasts appear, therefore, as the major component of the hematopoietic progenitor niche at the early stages of hematopoiesis that coincide with the establishment of the HSC compartment and being the producers of most diverse hematopoietic cytokines, such as KITL, CXCL12, IL-7, and Epo. As development progresses, they lose KITL, IL-7, and Epo and maintain low levels of CXCL12, which is primarily produced by mesenchymal cells, after E12.5. The exact chain of causality behind the localization of these cells would need further investigation: they might be the result of the environment being rich in chemokines and cytokines, they might be accumulating as the preferential neighbors of the hepatoblasts they rely upon, or both.

By E14.5, the submesothelial layer undergoes an overall reduction in thickness throughout the organ. This reduction is likely due to the recruitment of mesenchymal cells into the parenchyma to follow the expansion of the vascular network. Concurrently, mesenchymal cells increase their levels of *Cxcl12*. During this stage, KIT^+^ progenitors, primarily comprising erythroid cells, are still located close to the submesothelial region. Notably, these cells do not express CXCR4, suggesting that CXCL12 is not involved in shaping their distribution pattern. On the other hand, DP progenitors, which are responsive to CXCL12, are now randomly distributed. The neighbor analysis shows that DP cells do not depend on direct contact with SCs, suggesting that DP cells could respond to a CXCL12 gradient instead. Interestingly, a population marked in the Mds1^CreERT2^YFP mouse model that labels adult-type LSK cells, enriched in HSCs, does not preferentially associate with any of the stromal cells analyzed here, suggesting that they distribute randomly in the parenchyma. At E18.5, we see a discrete accumulation of CD45^+^ cells in the MCs/sub-MCs. The vicinity of the periphery is also putatively enriched in hematopoietic growth factors: CXCL12 produced by the dense layer of sub-MCs and KITL produced by MCs. These factors likely serve to attract DP cells toward the periphery and stimulate their differentiation into mature CD45^+^ cells, offering a plausible explanation for the distribution patterns observed at E18.5. Similarly, as development progresses, the regions near the mesothelium and the vessels, as they are highly enriched with CXCL12-producing cells, might be zones that retain the hematopoietic cells and induce differentiation, leading to the accumulation of mature cells in these regions at later stages.

### Limitations and perspectives

Our study presents several methodological constraints that warrant consideration. While our CD45 and KIT IF strategy enabled broad hematopoietic progenitor identification, it captured a heterogeneous population, potentially masking subset-specific spatial patterns. The use of the Mds1^CreERT2^YFP mouse model, though highly enriched for adult-type HSCs (Zhang et al., 2021), yielded limited cell labeling, precluding temporal analysis and comprehensive spatial mapping relative to the periphery, and confined our analysis to cell-type associations. Besides, only cells at E14.5, but not E12.5, could be labeled. The development of alternative genetic labeling approaches with higher recombination efficiency or multiplex labeling strategies is expected to enable a more detailed spatial analysis of HSC distribution within the FL microenvironment.

The Pyxidis analysis pipeline we developed demonstrates versatility for multidimensional datasets, enabling detailed cellular interaction analysis in complex biological systems. While our current implementation handles both single and multi-stack images effectively, we restricted our analysis to relatively small thick sections (∼50 μm) and mainly analyzed single-stacks. Integration with emerging whole-organ imaging technologies could expand our understanding of the three-dimensional organization of the FL niche and provide a more complete picture of cellular interactions across the entire organ. Such technical advances, combined with our established image analysis framework, would facilitate comprehensive characterization of the spatial architecture of the developing hematopoietic system.

## Materials and methods

### Mice

C57BL/6J mice were purchased from Envigo. *Flk1*-GFP mice were a gift from David Hardy (Hardy et al., 2019). Fixed *Cxcl12*-dsRed (Ding and Morrison, 2013) and *Kitl*-tdTomato (Buono et al., 2016) embryo samples were kindly provided by João Pedro Pereira and Marella de Bruijn, respectively. Mds1^CreERT2^ (Zhang et al., 2021) was supplied by Archibald Perkins (Rochester University, Rochester, NY, USA) and crossed to Rosa YFP reporter mice. 6- to 8-wk-old mice or timed pregnant females were used. Timed pregnancies were generated after overnight mating, and the following morning females with vaginal plugs were considered to be at E0.5. The day of birth was considered to be P0. Pregnant females were sacrificed by CO_2_ inhalation, and cervical dislocation was used as a confirmation method. E13.5 Mds1^CreERT2-YFP^ pregnant females were injected intraperitoneally with 47.7 μg/g body weight of OH-Tamoxifen and 23.8 μg/g body weight of progesterone and sacrificed 30 h later. For embryos older than E15.5 and pups, decapitation was performed. All animal manipulations were performed according to the ethics charter approved by the French Agriculture Ministry and to the European Parliament Directive 2010/63/EU.

### FC

#### Cell suspensions

For the isolation of non-hematopoietic populations, E12.5–E18.5 FLs were dissected under a binocular magnifying lens, recovered in Hanks’ balanced-salt solution (HBSS), supplemented with 1% fetal calf serum (FCS) (Gibco), cut into 1-mm^2^ pieces, and incubated with 0.05 mg/ml Liberase TH (Roche) and 0.2 mg/ml DNase I (Sigma-Aldrich) for 6–8 min at 37°C. Enzymatic treatment was stopped by the addition of cold HBSS with 1% FCS followed by centrifugation. FLs were depleted of hematopoietic cells (Ter119^+^CD45^+^KIT^+^CD71^+^) using MACS columns (Miltenyi Biotec) according to manufacturer instructions. Briefly, cells were incubated with biotinylated Ter119, CD45, Kit, and CD71 antibodies for 20–30 min at 4°C, washed twice, incubated with anti-biotin microbeads (Miltenyi Biotec) for 20 min at 4°C, and passed through an LS column (Miltenyi Biotec). The flow-through was collected, filtered with a 100 μm cell strainer (BD), and used for cell surface staining.

For the isolation of hematopoietic cells, E12.5–E14.5 FLs were dissected under a binocular magnifying lens, recovered in HBSS with 1% FCS (Gibco), and passed through a 26-gauge needle of a 1-ml syringe to obtain single-cell suspensions. Before staining, cell suspensions were filtered with a 100-μm cell strainer (BD).

For the isolation of BM non-hematopoietic populations, femurs and tibias were isolated from P7, P14, and P21 mice, and surrounding tissues were removed. Bones were flushed using a 26-gauge needle of a 1-ml syringe with HBSS with 2% FCS (Gibco). BM clumps were resuspended by gentling aspirating with a 19-gauge needle, filtered (100 μm), and kept on ice. Bones were crushed with a scissor in a way to maximize the internal bone surface area exposition and incubated with a digestion buffer (1 mg/ml Collagenase VIII and 0.2 mg/ml DNase I in HBSS with 2% FCS) for 15 min at 37°C with rotation. Cell suspensions were collected, filtered (40 μm), and kept on ice. Three rounds of digestion were performed, and cell suspensions collected from each were pooled together with the flushed ones. Enzymatic treatment was stopped by the addition of cold HBSS with 2% FCS followed by centrifugation. Cell suspensions were depleted of hematopoietic cells (Ter119^+^CD45^+^CD11b^+^B220^+^) using MACS columns (Miltenyi Biotec) as described before.

#### FC and cell sorting

Cell suspensions were stained with the antibodies listed in Table S1 for 20 min at 4°C. Non-hematopoietic FL cells were analyzed on a spectral cytometer ID7000 (SONY) as described (Peixoto et al., 2022) or sorted with a BD FACS Aria III (BD Biosciences). Non-hematopoietic BM cells were sorted with a BD FACS Aria III, and hematopoietic cells were analyzed on a custom BD LSR Fortessa (BD Biosciences) according to the guidelines for the use of FC and cell sorting (Cossarizza et al., 2019). Dead cells were excluded by the incorporation of propidium iodide. Data were analyzed with SONY ID7000 Analyzer, FlowJo software (v.10.8.2; BD Biosciences), and Jupyter Notebooks as described below.

#### Data analysis

FC files were screened for abnormalities (flow rate, signal acquisitions, and dynamic range) using a quality control plugin in FlowJo, FlowAI (Monaco et al., 2016). Only “GoodEvents” were considered for analysis. UMAPs were performed to dimensionally reduce the data (McInnes et al., 2020, *Preprint*) in FlowJo, on gated single, live, non-hematopoietic cells. For each stromal population defined, the corresponding channel values for the UMAP parameters were saved as .csv and plotted using Jupyter Notebooks (Kluyver et al., 2016).

### Imaging

#### FL sectioning, immunostaining, and optical clearing of thick sections

FLs from timed-pregnant females were collected directly into freshly prepared 2% paraformaldehyde fixative buffer (Thermo Fisher Scientific) and kept at 4°C overnight under rotation. After washing in PBS and removing surrounding tissues, FLs were immediately sectioned or shortly stored at 4°C until sectioning. Following FLs embedding in 4% low-melting agarose (Sigma-Aldrich), 150 μm frontal sections were performed using a Leica VT1200S vibratome. Sections were permeabilized and blocked with PBS containing 0.05% Tween-20 (AppliChem GmbH) (or 0.5% Triton-X 100 [Sigma-Aldrich] for nuclear staining) and 10% donkey serum (Sigma-Aldrich) for 1–2 h at room temperature. This solution was also used to dilute primary and secondary antibodies. Sections were incubated with primary antibodies for 2 h at room temperature and overnight at 4°C, and secondary antibodies for 2 h at room temperature, both in the dark and with rotation. Between incubations and after secondary antibody incubation, sections were washed repeatedly with PBS containing 0.05% Tween-20 and 3% NaCl (minimum 4× 15 min each). Sections were counterstained with DAPI (D9542; Sigma-Aldrich) for 2 h at room temperature and kept overnight in RapiClear 1.52 (SunJin Lab) for optical clearing. Sections were mounted in RapiClear and imaged on a Leica TCS SP8 using a PL APO 63×/1.40 oil immersion lens or APO 40×/1.40 oil immersion lens. Single-stacks or multi-stacks tile scans were acquired at 200 or 400 Hz, respectively, in bidirectional mode, at 12-bits and 1,024 × 1,024 resolution. Multi-stacks were acquired with z spacing of 0.3 μm. Table S2 compiles all information regarding image acquisition.

#### Image processing and nuclei segmentation

The FL images acquired in this study ranged in size from 2,682 × 2,826 to 7,686 × 8,669 × 117 pixels (Table S2), which corresponded to 60 Mb up to 50 Gb in memory needs. Because operations with big files are not suitable for analysis on graphics processing units (GPU) with 8 GB RAM, a preprocessing step was performed to reduce memory consumption. Each image was divided into small tiles—3,000 × 3,000 pixels for 2D images and 600 × 600 pixels × number of Z-stacks for 3D (see Table S2 for number of Z-stacks)—using the Python module Saucisson. For each tile, the nucleus was segmented, considering the DAPI signal, using the CellPose package (Stringer et al., 2021), in parallel on a GPU cluster. The segmented tiles, now with an additional channel corresponding to the mask of the segmented nuclei, were reconstructed to the original shape using Saucisson. At this point, we generated a tabular file with the position (x, y, and z coordinates) of each segmented cell and some attributes (signal intensity of each channel inside the mask; area/volume of the mask) using the package Griottes (Ronteix et al., 2022). To reduce artifacts created by incorrect segmentation of irregular nuclei, we filtered out segmented objects whose area/volume is inferior to 50 pixels^2^ or 3,000 pixels^3^, respectively.

#### Cell classification

Knowing the position of each cell, the next step is to distinguish the cell type. We employed two different methods for cell classification depending on the type of staining (nuclear or membrane). For nuclear staining (such as LHX2, FLK1, and HNF4α), the average signal intensity within the mask of the nucleus was computed using Griottes (Ronteix et al., 2022), a threshold was manually determined to distinguish between positive and negative cells, and its accuracy was visually confirmed.

For membrane staining (such as CD45, KIT), cells were classified using a deep learning-based neural network, which learns the fluorescence pattern around each nucleus. For that, the images were again deconstructed into small pieces, but now each one represents a single cell. Square images were generated for each detected nucleus with dimensions between 18 and 22 μm (80 × 80–120 × 120 pixels) and centered around the geometric center of the nucleus. The gray-scale pattern of the fluorescence inside this box was then analyzed by SqueezeNet Networks (Iandola et al., 2016, *Preprint*), using the transfer learning approach, and loaded, pretrained from PyTorch (Paszke et al., 2019, *Preprint*). Training and validation datasets were manually created for each staining using data from different images. The training was performed using a batch size of 32. As a result, an accuracy larger than 95% was achieved on validation datasets for each membrane staining, although some manual corrections in the classification were performed for images with lower staining contrast. A different network was trained for each marker to manage differences that appeared for the different membrane stainings. Manual verification of classification performances was done on 400 cells randomly selected for each membrane staining and each image (see number of false negative and positive cells in Table S2). For visual confirmation of the cell classification in the entire image, we used the graphical interface Napari, which allows us to overlay the original image with the resulting classification (Video 3).

#### Vascular network segmentation

For the identification and quantification of endothelial cells in the FL, we used *Flk1*-GFP mice and applied the threshold method for FLK1-GFP (nuclear staining) classification. For the segmentation of the vasculature, the CD146 signal was segmented by applying a threshold on the image and removing isolated pixels coming from the background. We obtained a mask for the CD146 signal from which we removed stainings coming from other organs using ImageJ. As CD146 is also staining MCs, we manually removed them to obtain a mask of the vessels. The distance between cells and the vasculature was then computed as the distance between each center of the nucleus and the closest positive pixel in the vessels’ mask. To compare with randomized FL, the vasculature is kept fixed, the rest of the cells are shuffled, and the distance is computed similarly (average taken over 10 randomizations).

#### Cell/region exclusion and selection of specific regions

Regions of the sections with pieces from other tissues or regions with lower staining quality were manually selected using the graphical interface Coloriage and excluded from analysis. AF cells were classified using the neural network approach, as described above, and excluded from analysis.

#### Network construction

After cell classification, we added to the data frame containing the positions and properties of the nuclei additional attributes related to the cell classification. A dot plot can then be generated where each dot represents a cell, and the color code identifies the cell phenotype. A “Delaunay” construction rule was applied to the data frame using Griottes to generate a connectivity network, considering a cutoff distance to avoid incoherent links (e.g., at the image’s periphery or vessels) (Ronteix et al., 2022). The returned object is a NetworkX graph (Hagberg et al., 2008), where the previously measured properties are node attributes (dots), while the neighborhood interactions and their attributes (length of the cell–cell contact) are encoded by the links.

#### Spatial analysis—Distance to target profiles and neighbor analysis

We conducted a statistical analysis of the cell distributions. To differentiate between relevant and statistically significant neighboring cell pairs and patterns of cell distributions that are not merely the result of a random chance, we created randomized spatial distributions for comparison. In this process, we shuffled the cell types on the network representation while preserving the total frequency of the cell types within the tissue (10 times). By labeling specific nodes in the graph as particular structures (periphery, PS, or a cell type), it is possible to calculate the distances of cells to those reference points. The minimum Euclidean distance between each cell in the network and the cells attributed to the structure of interest is computed, and the minimal distance is considered for each data point. Then the relative abundance of each cell type as a function of distance from the structure was determined (as represented in Fig. 5 a′). For random distributions, the distance to the periphery is recalculated for 10 repetitions of the reshuffling operation and averaged (Fig. 5 a′). When combining multiple images, the distribution profiles were normalized by the random profile (fold-change enrichment). In addition, we took advantage of the graph representation of the tissue to compute the composition of the neighborhood for a given cell type. As this depends on the cell type frequency in the tissue, we also compared the neighborhood composition, in terms of stromal populations, of hematopoietic cells to random spatial distributions. To do so, each stromal population (HNF4α, LHX2, CD146) is retained at its position, while all the other cells were subjected to reshuffling. For each image, we performed 10 randomizations and computed the frequency of contacts in the observed tissue and on average over the 10 randomizations. We then computed the ratio of these two quantities to obtain a fold change.

### Gene expression

#### Multiplex quantitative real-time (qRT-)PCR analysis of single-cells

Single cells were sorted directly into 96-well plates loaded with RT-STA Reaction mix (CellsDirect One-Step qRT-PCR Kit; Invitrogen, according to the manufacturer’s procedures) and 0.2× specific TaqMan Assay mix (see Table S3 for the TaqMan assays list) and were kept at −80°C at least overnight. For each subset analyzed, a control well with 20 cells was also sorted. Preamplified cDNA (20 cycles) was obtained according to the manufacturer’s note and was diluted 1:5 in TE buffer for qPCR. Multiplex quantitative PCR (qPCR) was performed using the microfluidics Biomark HD system for 40 cycles (Fluidigm) as previously described (Chea et al., 2016). The same TaqMan probes were used for both RT/pre-amp and qPCR. Only single cells for which at least two housekeeping genes could be detected before 20 cycles were included in the analysis.

Gene expression raw data of single cells was normalized with *Gapdh* and *Actb*. Heatmaps and hierarchical clustering were generated using R packages “pheatmap” and “Rphenograph” (Levine et al., 2015).

#### qRT-PCR bulk

Cells were sorted directly into the lysis buffer and mRNA was extracted with an RNeasy Plus Micro Kit (Qiagen). After extraction, mRNA was reverse transcribed into cDNA with PrimeScript RT Reagent Kit (Takara Bio), followed by qPCR with Power SYBR Green PCR Master Mix (Applied Biosystems). The primers used can be found in Table S4. qPCR reactions were performed on a Quantstudio3 thermocycler (Applied Biosystems), gene expression was normalized to that of *Actb*, and relative expression was calculated using the 2^−ΔCt^ method.

### Transwell migration assays

FL KIT^+^ and DP cells were prepared as described above for cell sorting. For sorting of E14.5 cells, lineage depletion (Ter119, CD19, B220, Gr1, CD3, CD4, CD8a, NK1.1, and CD11c) was performed using magnetic LS columns (Miltenyi Biotec). Dual-chamber chemotaxis assays were performed using 24-well plates with 5-μm pore size inserts (Cat. PTMP24H48; Sigma-Aldrich), as previously described (Christensen et al., 2004). CXCL12a (Cat. 460-SD-050; R&D Systems) was added to the lower chamber at defined concentrations, and 100 μl of a cell suspension (1 × 10^6^ cells/ml) of KIT^+^ or DP cells were placed in the upper chamber. Cells were allowed to migrate for 2 h at 37°C. A known quantity of fluorescent beads was added to the lower chamber for normalization of migrated progenitors. Migrated cells were collected from the lower chamber and analyzed by FACS to enumerate migrated progenitors.

### Spheroid assay

E14.5 cell stromal cell suspensions were obtained by depletion of KIT^+^, Ter119^+^, CD71^+^, and CD45^+^CD31^+^ cells, using a MACS column. For the isolation of hepatoblasts, we added Gp38 and PDGFRα antibodies to the depletion mix. 1,000 stromal cells were cultured in ultra-low attachment round bottom plates in OPTI-MEM supplemented with 5% heat-inactivated FCS, 1% Pen/Strep, and 5 × 10^−4^M β2-ME, 10 μg/ml insulin, 30 ng/ml IGF2, and 50 ng/ml EGF with 1,000 sorted TER119^−^CD71^−^CD45^+^ hematopoietic cells. ACK2 antibody (Nishikawa et al., 1991) was added at the beginning of the culture at a concentration of 1.25 µg/ml in 200 µl per well. This concentration was determined in preliminary tests for no interference with the staining of KIT^+^ cells with another KIT-specific antibody, 2D8. This concentration was also not depleting KIT^+^ when added to BM cells incubated for 1 h at 37°C. Individual spheroids were dissociated using undiluted Accutase (Thermo Fisher Scientific) for 10–20 min at 37°C under shaking. Efficient spheroid dissociation was monitored under the microscope.

### Statistical analysis

The results are presented as mean ± SD. Statistical analyses were conducted using GraphPad Prism or Jupyter Notebooks, with the specific tests employed indicated in the figure legends.

### Online supplemental material

Fig. S1 shows the gating strategy for isolation of FL hematopoietic and stromal cell types, along with heatmaps of single-cell gene expression profiles. Fig. S2 examines the composition of CD45^+^, DP, and KIT^+^ cell populations at E12.5 and E14.5. Fig. S3 provides validation and quality control data for the image analysis, including classification accuracy for KIT and CD45 markers, as well as details on AF cell exclusion and vessel network segmentation. Fig. S4 shows cytokine expression patterns across FL development (E12.5–E18.5) in stromal populations and comparison with BM stromal populations at different stages. Fig. S5 shows the neighbor analysis at E14.5, *Cxcr4* gene expression from different progenitor cells, and additional representative IF images of *Cxcl12*-dsRed mice at E14.5. Video 1 shows the intricate vascular and perivascular network of the E18.5 FL. Video 2 shows ALCAM displays a widely distributed expression pattern across FL populations. Video 3 shows visual confirmation of the segmentation and classification using Napari. Video 4 shows the pattern of expression of *Cxcl12* in *Cxcl12*-dsRed reporter mice at E12.5. Video 5 shows the pattern of expression of *Kitl* in *Kitl*-tdTomato reporter mice at E12.5. Table S1 indicates all the antibodies used in this study. Table S2 compiles the metadata of all images analyzed and displayed in this manuscript. Table S3 indicates the TaqMan assays used for the single-cell multiplexed transcriptomic analysis. Table S4 indicates the primers used for qRT-PCR.

## Supplementary Material

Table S1 list of antibodies used for FC and IF.

Table S2 shows image analysis metadata.

Table S3 shows TaqMan assays used for single-cell multiplexed qRT-PCR.

Table S4 list primers used for qRT-PCR.

## Data Availability

All data needed to evaluate the conclusions in the paper are present in the paper and/or the supplementary materials. All raw data are available in the open repository Zenodo with the following unique identifiers: Pyxidis image example dataset: https://doi.org/10.5281/zenodo.7867024. CSV files for the image analysis results of the paper: https://doi.org/10.5281/zenodo.7866577. Images of Mds1 stem cells: https://doi.org/10.5281/zenodo.14287721. Gene expression data (bulk and single cell): https://doi.org/10.5281/zenodo.7867187. FACS data FL: https://doi.org/10.5281/zenodo.7867211. FACS data co-cultures: https://doi.org/10.5281/zenodo.14287502. The analysis of the FL network images was conducted using Python on Jupyter Notebooks. All the relevant notebooks are freely accessible on the project GitHub repository (https://github.com/BaroudLab/Pyxidis). Different packages have been custom-made for the analysis of the FL images during preprocessing and data analysis. All these functionalities (Saucisson, Coloriage) have been incorporated into the main GitHub for easier use and installation. This repository contains a step-by-step tutorial to run the entire image analysis pipeline and data analysis previously described.
